# Positive wellbeing and resilience following adolescent victimisation: An exploration into protective factors across development

**DOI:** 10.1002/jcv2.12024

**Published:** 2021-07-15

**Authors:** Jessica M. Armitage, Rui Adele Wang, Oliver S. P. Davis, Philip Collard, Claire M. A. Haworth

**Affiliations:** ^1^ School of Psychological Science University of Bristol Bristol UK; ^2^ School of Economics, Finance and Management University of Bristol Bristol UK; ^3^ Population Health Sciences Bristol Medical School Bristol UK

**Keywords:** ALSPAC, peer victimisation, protective factors, resilience, wellbeing

## Abstract

**Background:**

Not all victims of bullying go on to develop problems with their mental health. To understand factors that may confer resilience, many have explored the moderating role of protective factors in relation to mental illness. No study to date, however, has considered moderators of adult wellbeing following victimisation. We explore 14 protective factors and test whether these promote good adult wellbeing in addition to prevent mental illness following victimisation. In doing so, we aimed to understand how positive mental health and resilience can be promoted.

**Methods:**

Data were derived from the Avon Longitudinal Study of Parents and Children. Participants were assessed for wellbeing and depressive symptoms at age 23, as well as victimisation in adolescence, and protective factors across development. Protective factors were categorised into individual‐, family‐ and peer‐level, and included factors like social skills, perceived school competence, and relationships with family and peers. The moderating role of the protective factors were examined using interactive regression models.

**Results:**

Perceived scholastic competence was the only factor that mitigated some of the negative effects of victimisation. Individuals with higher perceptions of scholastic competence had higher wellbeing in adulthood than victims with lower perceptions of competence. No protective factors positively moderated life satisfaction or the risk of depressive symptoms; although findings suggest that friendships in late adolescence may be protective for individuals exposed to less frequent victimisation.

**Conclusions:**

Our study is the first to explore a wide range of protective factors in predicting adult wellbeing following victimisation. We identify factors involved specifically in supporting wellbeing but not in reducing the risk of depression. Findings suggest that interventions aimed at increasing perceptions of scholastic competence in childhood may help to support more positive wellbeing in adulthood.

## BACKGROUND

Although peer victimisation is a major public health concern, associated with poorer physical and mental health (Wolke & Lereya, 2015), findings have revealed substantial resilience among victims. Peer victimisation occurs when an individual is repeatedly exposed to discomfort from another peer's behaviour. Consequences include an increased risk for depression and anxiety; however, this risk is attenuated over time (Singham et al., 2017), with around 15% of victims depressed by early adulthood (Bowes et al., 2015). This suggests that most do not go on to develop clinical depression. Such individuals provide the opportunity to explore predictors of resilience to inform best practice for interventions.

**Key points**

- Peer victimisation not only predicts an increased risk of mental illness, but also poorer wellbeing in adulthood
- Victims who hold higher perceptions of scholastic competence in childhood have greater wellbeing in adulthood than victims who reported lower scholastic competence in childhood
- The protective effects of late adolescent friendships on adult mental health and wellbeing are less apparent among those exposed to more frequent victimisation
- School‐based interventions aimed at increasing perceptions of scholastic competence may help to reduce the burden of victimisation on wellbeing in later life

Resilience is not clearly defined; however, many view it as the ability to adapt successfully despite adversity (Ungar et al., 2013). Resilience is often investigated through the study of protective factors that positively moderate responses to adversity (Rutter, 1985). Explorations into protective factors following victimisation have focused on internalising and externalising problems (Sapouna & Wolke, 2013). A systematic review of such findings identified common protective factors in childhood and adolescence at the individual‐, family‐ and peer‐level (Ttofi et al., 2014). Few, however, have considered how they impact wellbeing.

Wellbeing refers broadly to optimal psychological functioning (Ryan & Deci, 2001). Some refer to hedonic and eudaimonic wellbeing, whereby hedonic relates to happiness and eudaimonic describes meaning and self‐realisation (Ryan & Deci, 2001). Mental wellbeing is often used to encompass both dimensions and has been shown to predict various positive outcomes, including greater physical health and interpersonal relationships (Regan et al., 2016). Positive wellbeing is also associated with fewer mental health problems; however, correlations between wellbeing and mental illness are moderate (Haworth et al., 2017), meaning individuals may not show signs of a mental illness but still be unhappy. Indeed, individuals who avoid depression following victimisation have poorer wellbeing than their non‐victimised counterparts (Armitage et al., 2021).

Although the large literature linking victimisation to mental illness has highlighted the need to target victims of bullying, it is also accepted that eradicating victimisation is unlikely (Arsenault, 2017). Efforts to support victims must therefore explore how resilience can be fostered. To do this, it is necessary to consider how wellbeing can be promoted and mental illness prevented. No study, however, has yet explored moderators of adult wellbeing among victims of bullying. Below we review findings on the impact of protective factors in reducing mental illness following victimisation to inform those that may promote wellbeing.

### Individual‐level protective factors

Individual‐level factors identified as protective following victimisation include a high self‐esteem, good social skills and good school performance (Ttofi et al., 2014). Self‐esteem describes the orientation towards oneself, which is used to evaluate self‐worth, referring to self‐liking and self‐competency, feeling capable (Tafarodi & Swann, 1995). Studies have suggested self‐worth is more important for mental health following victimisation than self‐competency (Soler et al., 2013). Such findings, however, derive from research on various victimisation experiences, not all of which were inflicted by peers. Studies assessing overall self‐esteem in relation to peer victimisation specifically have demonstrated significant moderating effects on emotional adjustment (McVie, 2014), with victims holding a more favourable view of themself at a lower risk of depression in adolescence (Sapouna & Wolke, 2013). No study, however, has considered the impact of self‐esteem in moderating the effects of peer victimisation on adult mental illness or wellbeing.

Research exploring the role of factors in predicting the risk of adult mental illness have, however, revealed that adolescents with higher social skills are less than half as likely to report depression following victimisation (Vassallo et al., 2014). Victims who stated that they understood the work in their classes were also at lower risk, with rates of depression dropping by 16% (Vassallo et al., 2014). Similar longitudinal research revealed the prevalence of victims with depressive symptoms in adulthood drops from 23.1% to 6.9% among low versus high achievers (Hemphill et al., 2014). These findings, however, rely on single‐item assessments of victimisation. The studies also dichotomised factors into protective or non‐protective categories, resulting in small samples of participants. Results should therefore be interpreted with caution.

Overall, no study has explored whether the benefits of individual attributes extend beyond preventing mental illness to promoting wellbeing.

### Family‐level protective factors

Positive relationships between a child and their family have also been suggested as key to preventing maladjustment following peer victimisation (Stadler et al., 2010). Studies have shown that a positive home environment, as well as high maternal warmth, protect victims against emotional problems in childhood (Bowes et al., 2010), with similar findings reported among adolescents with high parental support (Stadler et al., 2010). The extent to which these relationships continue to protect against mental illness in adulthood when victims are less likely to live with family members remains unexplored. Much like the individual‐level protective factors, it is also not yet known whether family influences extend to promoting wellbeing.

### Peer‐level protective factors

Beyond family relationships, peer relationships have also been suggested to play a role in promoting resilience to victimisation, although research is mixed. While some evidence shows that having more supportive friends protects victims against internalising problems (Papafratzeskakou et al., 2011), others have found that this effect is not specific to victims, but equally important for victims and non‐victims (Averdijk et al., 2014). Findings have also revealed that friendships may intensify the risk of mental illness, with depression rates greater among victims who report high peer attachment compared to non‐victims with high attachment (Vassallo et al., 2014). Together these findings emphasise the need for further exploration into the role of peers.

Just one study has considered the impact of peers on wellbeing following victimisation. Flaspohler et al., (2009) show that peer support may be protective; however, only adolescent life satisfaction was assessed. No study has explored the longitudinal and moderating role of protective factors on wellbeing following victimisation, or gone beyond the impact on life satisfaction. Doing so is crucial as victimisation not only increases the risk of mental illness but also negatively impacts adult wellbeing (Armitage et al, 2021).

### Protective factors across time

To improve our understanding of resilience, it is necessary to study protective factors over time (Ungar et al, 2013). Recent findings have shown that among victims of childhood adversity, protective factors in earlier adolescence are more successful in reducing distress compared to those in later adolescence (Fritz et al., 2019). No study, however, has explored protective factor changes in relation to victimisation, or considered their role prior to victimisation. Studying early precursors of resilience could enable the detection of time‐independent protective factors (Fritz et al., 2018). These could prove essential to preventative programmes by bolstering resilience to future adversity.

### Current study

We investigate for the first time, the degree to which factors promote adult wellbeing and protect against mental illness after victimisation. We go beyond current findings focused on one stage of development to include protective factors from childhood through to late adolescence. We also explore the cumulative effects of factors at the individual‐, family‐ and peer‐level.

## METHODS

### Participants

Participants were from the Avon Longitudinal Study of Parents and Children (ALSPAC), a prospective cohort in the United Kingdom (Boyd et al., 2013). Pregnant women in the former Avon area with an expected delivery date between April 1991 and December 1992 were enrolled (Fraser et al., 2013). The cohort consisted of 14,062 live births but has increased to 14,901 children with further recruitment (Northstone et al., 2019). Data from 22 years were collected and managed using REDCap, hosted at the University of Bristol (Harris et al., 2019). Please note that the study website contains details of all the data that are available through a fully searchable data dictionary and variable search tool: http://www.bristol.ac.uk/alspac/researchers/our‐data/

### Subsample

At 13 years, participants completed a victimisation assessment (*n* = 6529), of which 3015 (46.2%) also completed a measure of wellbeing at age 23. Data were taken from these individuals and those who also completed the depressive symptoms measure at age 23. This ensured fair comparisons between models predicting wellbeing and depressive symptoms. Included individuals also had information relating to their socioeconomic status (SES) in addition to the protective factors (see Figure S1 and Table S1 for information about attrition). In total, 949 participants had complete data for victimisation, wellbeing, depression and all protective factor measures. To maximise available data and avoid the potential for bias that may arise from using complete cases, analyses explored protective factors separately. While this resulted in samples ranging from 1712 to 2398, comparisons revealed no differences in sex, victimisation, SES or mental health (see Table S2). Correlations between protective factors were also low (see Tables S3 and S4), suggesting it is unlikely that sub‐samples will influence results. We did, however, explore the impact of attrition by imputing missing values. This was done using sociodemographic factors associated with missingness in ALSPAC. The list of variables used for imputation are in Table S5.

### Measures

Details of the variables are in Table 1, including who was assessed and when, the number of items on each scale and how these were scored, as well as their internal consistency (Cronbach's alpha).

**TABLE 1:** Description of study variables

Construct	Number of items	Sample item	Scoring	Composite creation	Higher score represents	Cronbach's alpha	Completed by	Age
Peer victimisation	9	‘Frequency someone tricked teenager’	0–4 (‘never’–‘>1/week’)	Sum	More victimisation	0.73	Participant	12.5
Mental wellbeing	14	‘I've been feeling relaxed’	0–4 (‘none of the time’–‘all of the time’)	Sum	Higher wellbeing	0.93	Participant	23
Depressive symptoms	13	‘I felt miserable or unhappy’	0–3 (‘not at all’–‘true’)	Sum	More symptoms	0.91	Participant	23
Life satisfaction	5	‘I'm satisfied with my life’	1–7 (‘strongly disagree’–‘strongly agree’)	Sum	Higher life satisfaction	0.89	Participant	23
Covariates								
SES	1	‘What is your present job? If not working, what was your most recent job?’	Nine social occupational classifications	Mean	Lower SES	‐	Parent	18 weeks gestation
SES	1	‘What is your highest educational qualification?’	0–5 (‘degree’–‘none’)	Mean	Lower education	‐	Parent	32 weeks gestation
Protective factors: Individual level								
Scholastic competence	6	‘Do well at schoolwork’	1–4 (‘yes, really like me’–‘no, not at all like me’)	Sum	Higher competence	0.88	Participant	8
Global self‐worth	6	‘Happy with self as a person’	1–4 (‘yes, really like me’–‘no, not at all like me’)	Sum	Higher self‐worth	0.89	Participant	8
Social skills	12	‘Does not realise when others are upset’	0–3 (‘not true’–‘true’)	Sum	Higher social skills	0.88 (all)	Mother	7.5, 13, 16
Self‐perceived academic ability	6	‘Rating of maths ability’	1–5 (‘very good’–‘not good at all’)	Mean	Higher perceived ability	0.54	Participant	13
Protective factors: Family‐level								
Family support and relationships	5	‘How close do you feel to your parents?’	1–4 (‘very close to at least one’–‘Not close at all to either’)		Closer relationship	‐	Participant	17.5
Protective factors: Peer‐level								
Friendships	5	‘Believes friends understand them’	1–4 (‘most of the time’–‘not at all’)		Positive friendship	0.50	Participant	8, 12.5, 17.5
						0.46		
						0.74		

Abbreviation: SES, socioeconomic status.

### Peer victimisation

Peer victimisation was measured using the previously validated, nine‐item Bullying and Friendship Interview Schedule (Wolke et al., 2001). Correlations between direct and indirect experiences were moderate (*r* = 0.52), therefore scores were combined. The overall measure ranged from 0 to 25 (*M* = 1.81, *SD* = 2.75), with 0 representing those who had never been victimised. A three‐level ordinal variable derived from these scores showed that 46.4% of adolescents were never victimised, 36.1% were occasionally victimised (scored 1–3) and 17.5% were frequently victimised (scored 4 or more). Owing to high amounts of positive skew, analyses were carried out using continuous scores that were log transformed (after adding a constant of 1 to accommodate scores of zero). Results using untransformed scores can be found in Tables S6 and S7.

### Wellbeing

Our primary wellbeing outcome was the Warwick‐Edinburgh Mental Well‐Being Scale (Tennant et al., 2007). This 14‐item scale is widely used for policy evaluations and was chosen to ensure hedonic and eudaimonic wellbeing were assessed. Follow‐up analyses used scores from the five‐item Satisfaction with Life Scale (Diener et al., 1985) to allow comparisons with the only similar study to date on protective factors in relation to wellbeing (Flaspohler et al., 2009). Correlations between life satisfaction and mental wellbeing were high (*r* = 0.66).

### Depression

In further analyses, we used the 13‐item shortened version of the Moods and Feelings Questionnaire (Angold et al., 1995) to assess depressive symptoms. This allowed us to test possible distinctions between protective factors involved in mental illness and wellbeing. The reliability of these measures is reported in Table 1.

### Protective factors

#### Individual‐level

Scholastic competence and self‐worth were assessed using a shortened version of Harter's Self Perception Profile for Children (Harter, 1985). This has good test–retest stability (Muris et al., 2003). We examine scholastic competence and self‐worth separately as findings suggest distinct effects following victimisation (Soler et al., 2013). Self‐esteem was not available in adolescence; however, childhood self‐esteem is more appropriate as later measures may be negatively influenced by victimisation (Skues et al., 2005).

The Social Communication Disorder Checklist assessed social skills. This measure has excellent internal consistency and test–retest reliability (Skuse et al., 2005). Scores in our study had a negative skew greater than 1, therefore analyses were repeated using a reflected and log‐transformed variable, results of which are in Table S8. We present the untransformed results to enable comparisons with the other untransformed factors.

Self‐perceived academic ability was captured using ratings of English, Maths, Science, Art, ICT and Sport ability. We focus on self‐reported ratings to maximise the data available and ensure findings align with previous research (Vassallo et al., 2014).

#### Family‐level

Family relationships were assessed during a short‐structured interview. Five questions were used to assess parental closeness (How close do you feel to your parents?), sibling closeness (How close do you feel to your siblings?), family support (How easy do you find it to discuss problems with people in your family?), family involvement (How often do you do things together as family?) and family cohesion (How well have you been getting along with the family?).

#### Peer‐level

Friendships were assessed using a structured interview that included five questions from the Cambridge Hormones and Moods Project Friendship Questionnaire (Goodyer et al., 1990): ‘Are you happy with the number of friends you've got?’, ‘How often do you see your friends outside of school?’, ‘Do your friends understand you?’, ‘Do you talk to your friends about problems’, ‘Overall, how happy are you with your friends?’.

### Covariates

Analyses controlled for sex and SES because of differences in relation to wellbeing (Kaplan et al., 2008). As indices of SES, we used parental reports of their educational qualifications and occupational status. Both items were summed, generating scores ranging from 2 to 11 (*M* = 6.08, *SD* = 2.00).

### Statistical analysis

To investigate whether protective factors moderate the mental wellbeing of individuals following victimisation, we ran linear regression models that included an interaction term (log‐transformed victimisation scores by each protective factor). This allowed us to determine the main and interactive effects of victimisation and the protective factors.

Follow‐up analyses exploring the interactive effects of the protective factors on life satisfaction also used linear regression models, while negative binomial regression models were used for analyses predicting depressive symptoms to control for negative skew. We chose the negative binomial model over the Poisson model as scores did not have identical parameters for the mean and variance (*M* = *7*.03, *σ* ^*2*^ = 36.60). The combined impact of protective factors at different levels (individual, family, and peer) were investigated using principal components analysis (PCA). Loadings of the principal components on each protective factor can be found in Table S9. Kaiser's criterion of 1 was used to explore the moderating role of components on wellbeing, life satisfaction and depression. We subsequently used a hierarchical PCA to assess the cumulative impact of factors across the different levels. To facilitate interpretability, all protective factor measures were transformed into z‐standardised variables. Where longitudinal data on the protective factors were available, analyses explored the importance of timing by comparing protective effects before, during and after victimisation.

Analyses were run in R Studio version 4.05 (R Core Team, 2021). The ‘MASS’ package (Venables & Ripley, 2002) was used for the negative binomial regressions and the ‘rsq’ package (Zhang, 2017) to generate R‐squared estimates. For the PCA, we used the ‘prcomp’ function within the ‘stats’ R package. Multiple imputation was conducted using the Chained Equations (MICE) package (Van Buuren & Groothuis‐Oudshoorn, 2010). Based on Rubin's rules (Little & Rubin, 2014), we ran 60 imputations. To control for the probability of making a Type I error on multiple comparisons, Benjamini–Hochberg False Discovery Rate was used. This allows for the non‐independence of repeated tests (Benjamini & Hochberg, 1995).

## RESULTS

### Descriptive data

Across sub‐samples, 63.5% of participants were female and up to 17.1% experienced frequent victimisation, defined as scoring above 4 on our victimisation measure. Wellbeing scores averaged 49.31 (range 14–70) and were significantly higher among non‐victims compared to those exposed to occasional or frequent victimisation (Table S10).

### Main and interactive effects

All protective factors, excluding childhood scholastic competence, were positively associated with wellbeing at the population level (Table 2) and remained so after correction for multiple testing.

**TABLE 2:** Impact of victimisation (log‐transformed), protective factors and their interaction on wellbeing

	*N*	Protective factor	Victimisation	Interaction	
		*β* (95%C.I.)	*β* (95%C.I.)	*β* (95%C.I.)	Δ*R* ^2^
Individual‐level					
Scholastic competence	2302	0.10 (−0.42, 0.62)	−1.3 (−1.8, −0.83)***^,^ ^a^	0.63 (0.15, 1.1)* ^,^ ^a^	0.58%
Global self‐worth	2296	0.69 (0.16, 1.2)*^,^ ^a^	−1.3 (−1.7, −0.78)***^,^ ^a^	0.25 (−0.22, 0.73)	0.88%
Childhood social skills	2330	1.2 (0.56, 1.8)***^,^ ^a^	−1.3 (−1.7, 0.8)***^,^ ^a^	−0.05 (−0.55, 0.45)	1.0%
Adolescent social skills	2339	0.95 (0.38, 1.5)**^,^ ^a^	−1.3 (−1.7, −0.78)***^,^ ^a^	0.39 (−0.09, 0.88)	1.7%
Late adolescent social skills	2092	0.99 (0.42, 1.6)***^,^ ^a^	−1.1 (−1.6, −0.64)***^,^ ^a^	0.38 (−0.11, 0.87)	2.0%
Academic ability	2360	1.3 (0.08, 1.8)***^,^ ^a^	−1.3 (−1.7, −0.08)***^,^ ^a^	0.16 (−0.30, 0.62)	2.4%
Family‐level					
Closeness to parents	1838	1.3 (0.76, 1.9)***^,^ ^a^	−1.1 (−1.6, −0.53)***^,^ ^a^	0.01 (−0.52, 0.54)	2.0%
Closeness to siblings	1712	1.6 (1.0, 2.2)***^,^ ^a^	−1.1 (−1.6, −0.55)***^,^ ^a^	−0.41 (−0.95, 0.15)	2.1%
Family support	1833	1.5 (0.97, 2.1)***^,^ ^a^	−1.0 (−1.5, −0.51)***^,^ ^a^	0.00 (−0.52, 0.51)	2.8%
Family involvement	1824	0.74 (0.19, 1.3)**^,^ ^a^	−1.1 (−1.6, −0.56)***^,^ ^a^	0.40 (−0.12, 0.92)	1.4%
Family cohesion	1838	1.4 (0.78, 1.9)***^,^ ^a^	−0.90 (−1.4, −0.38)***^,^ ^a^	0.11 (−0.41, 0.62)	2.5%
Peer‐level					
Childhood friendships	2303	0.62 (0.08, 1.2)*^,^ ^a^	−1.3 (−1.8, −0.83)***^,^ ^a^	−0.05 (−0.53, 0.42)	0.30%
Adolescent friendships	2398	0.91 (0.39, 1.4)***^,^ ^a^	−1.4 (−1.6, −0.65)***^,^ ^a^	0.04 (−0.40, 0.48)	0.98%
Late adolescent friendships	1811	2.5 (1.9, 3.0)***^,^ ^a^	−0.98 (−1.5, −0.45)***^,^ ^a^	−0.75 (−1.3, 0.24)** ^,^ ^a^	5.2%

*Note:* Δ*R* ^2^ represents the incremental *R* ^2^.

^a^ FDR.

***p < 0.001, **p < 0.01, *p < 0.05.

**FIGURE 1:**
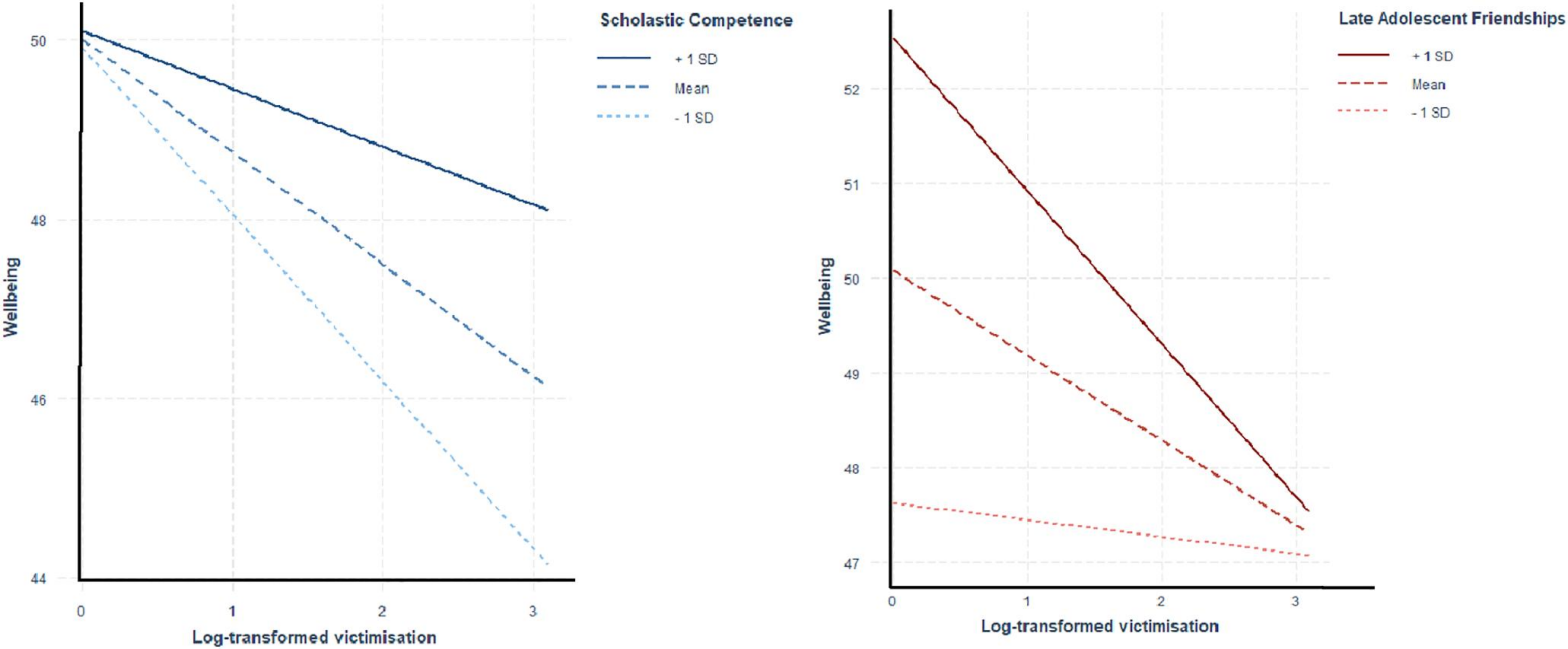
Interactive effects of victimisation and protective factors on wellbeing

The interaction models revealed significant moderating effects of perceived scholastic competence (Table 2). As victimisation scores increased, individuals were more likely to have higher wellbeing if they scored higher on the scholastic competence scale (Figure 1). This protective factor explained 0.58% of the variance in wellbeing after accounting for victimisation and the covariates (see Table S11 for full results, including *p*‐values). Interactions were also observed between victimisation and friendship scores in late adolescence. Such interactions accounted for 5.2% of the variance in wellbeing (Table 2). Plots of the interactions show that individuals with lower victimisation scores experienced significantly higher wellbeing if they also reported more positive friendships (Figure 1). However, for individuals exposed to more victimisation, having more positive friendships did not significantly alter wellbeing. These interactions remained after correction for multiple testing. Analyses using the untransformed victimisation scores and imputed data set produced highly consistent results (Tables S6 and S12), with confidence intervals that overlapped. For analyses using the imputed data, significant interactions were also observed with other factors, reflecting the increase in power gained from a larger data set.

### Follow‐up analyses

Models predicting life satisfaction revealed similar results for the main effects; however, we found no evidence of moderation (Tables S7 and S13). For analyses predicting depressive symptoms, all protective factors were negatively associated at the population level, and interactions were observed with friendships in late adolescence (Tables S7 and S13). This interaction, however, did not remain after correction for multiple testing.

Results using variables from the PCA are presented in Tables S14 and S15. Interactions were observed between victimisation and the first individual‐level component in predicting wellbeing (accounting for 4.1% of variance) and life satisfaction (4.9% of variance), but neither the family‐ or peer‐level components, or the combined factor generated from the hierarchical PCA, showed interactive effects.

## DISCUSSION

Our study is the first to consider moderators of adult wellbeing following peer victimisation. Findings suggest that victims who hold higher perceptions of scholastic competence in childhood have greater wellbeing in adulthood. These protective effects were specific to mental wellbeing and were not observed for life satisfaction or depression, reinforcing the need to consider both positive and negative mental health to understand resilient functioning.

### Individual‐level protective factors

The moderating effects of perceived scholastic competence could reflect an increased participation in school. Victims with greater perceptions of ability may become more involved in academic activities to distract them from problematic peers. This involvement is likely to increase school connectedness and enjoyment, and may provide a buffer against negative effects of victimisation on wellbeing. It is possible that perceived scholastic competence also increases academic attainment, which itself is related to increased wellbeing (Nikolaev, 2018). However, we believe this is less likely as no moderating effects were found using adolescent academic ability.

Adolescents in our study rated their ability in specific school subjects, while our childhood measure assessed overall competency with schoolwork. This latter measure is less affected by specific abilities and is more commonly used to determine self‐esteem. Increases in self‐esteem about schoolwork may help boost student morale which would otherwise have been damped by victimisation. Future research should explore whether adolescent self‐esteem moderates the impact of victimisation on adult wellbeing to provide further insight into the path by which perceived scholastic competence may increase wellbeing among victims.

Other individual‐level factors in our study produced similar effects to those found previously in adulthood (Vassallo et al., 2014), with factors like social skills predictive of fewer problems at the population level, but no moderating effects for victims. Unlike previous findings which used binary measures of victimisation, we used continuous scores to avoid arbitrary cut‐offs and small samples. This enabled sufficient power to rule out any large moderating effects.

### Peer‐level protective factors

Our findings extend previous interactive effects of peers in early adolescence (Flaspohler et al., 2009) by demonstrating moderating effects of peers in later adolescence. In particular, we show that while peers exert protective effects on adult wellbeing, this diminishes as individuals are exposed to more victimisation. Past findings were based on groups of victims or non‐victims, meaning detecting subtle differences between those exposed to varying frequencies of victimisation was not possible. Our findings suggest that the support individuals receive following repeated victimisation may not be sufficient to foster resilience. This is likely due to the dose‐response effect between victimisation and mental health (Armitage et al., 2021). Peers may be protective but only to experiences less detrimental to mental health. It is also possible that results reflect the type of friendships formed. Individuals are more likely to befriend those with similar levels of internalised distress (Hogue & Steinberg, 1995). For individuals subjected to frequent victimisation, such friends may co‐ruminate and heighten stress. High levels of support from these peers may also be coupled with negative aspects of friendship like jealousy. This may prevent positive buffering effects on mental health.

### Family‐level protective factors

Our results do not provide support for family‐level protective factors. Previous studies exploring the protective role of families focused on childhood (Bowes et al., 2010). We explored the role of families during late adolescence in predicting wellbeing among young adults, suggesting protective effects may lessen as victims reach adulthood. This is supported by findings that show parental support is most effective in buffering against internalising problems among younger adolescent victims (Stadler et al., 2010).

### Protective factors across development

Our study provides unique insight into the importance of timing of protective factors and suggests that those most beneficial to adult wellbeing are likely to be in place prior to victimisation. The presence of such factors may be more accessible than those that occur after victimisation and may help to establish additional protective factors. In the current study, individuals with higher perceptions of competence may also have had a greater capacity to deal with day‐to‐day stresses early on, making them less susceptible to the adverse effects of later victimisation. Identifying such protective factors in childhood could thus increase chances of resilience by ensuring early and potentially targeted intervention.

Resilience within our study was inferred by comparing the wellbeing of victims of frequent bullying exposed to different levels of protective factors. While we had sufficient power (>80%) to detect small moderating effects (see supplementary), comparisons were based on approximately 17% of individuals reporting frequent victimisation. While this is larger than previous studies (Vassallo et al., 2014), cohorts with more victims may detect additional moderating effects. Studies also including longitudinal assessments of wellbeing may reveal relevant protective factors at different time points.

Other limitations are that due to data unavailability, some protective factors relied on self‐reports. Self‐reports may be biased among victims with negative cognitions. Future research should thus attempt to distinguish between perceived and actual support to provide a more objective assessment. Further studies should also consider support from romantic partners which was not explored due to data unavailability. Our longitudinal cohort may also limit the generalisability of results as participants with data available for victimisation and wellbeing may be more likely to have fewer mental health problems compared to those missing. However, previous studies have shown this to have a marginal effect on study estimates in ALSPAC (Wolke et al., 2009). We also replicated the findings using an imputed data set that used depressive symptoms as a predictor of missingness. Reverse causality should also be considered as it is possible that no moderating effects were observed for other factors, such as adolescent academic ability, due to the impact that victimisation may have, for example, on school attendance.

Overall, our study highlights the importance of investigating protective factors before and after victimisation, as well as to varying frequencies of victimisation. Findings imply there may be distinct factors involved in moderating wellbeing and the risk of depression among victims exposed to different levels of victimisation. Multiple interventions may therefore be necessary to both promote wellbeing and prevent mental illness among victims of bullying. School‐based interventions aimed at increasing perceptions of scholastic competence in childhood could be an efficient means of reducing the burden of victimisation.

## CONFLICT OF INTEREST

The authors have declared that they have no competing or potential conflicts of interest.

## ETHICAL APPROVAL

This study was approved by the ALSPAC Ethics and Law Committee and the Local Research Ethics Committees. Full details of the ethics committee approval references can be found online (http://www.bristol.ac.uk/alspac/researchers/research‐ethics/). Informed consent for the use of data collected via questionnaires and clinics was obtained from participants who have the right to withdraw their consent at any time.

## AUTHORS CONTRIBUTIONS

Jessica M. Armitage defined the research question with Claire M. A. Haworth. Funding acquisition and data collection for the wellbeing material in ALSPAC were performed by Claire M. A. Haworth. Data preparation, analysis, and investigation were performed by Jessica M. Armitage. Interpretations of the data were made by Jessica M. Armitage, Rui Adele Wang, Oliver S. P. Davis, Philip Collard and Claire M. A. Haworth. The original draft of the manuscript was written by Jessica M. Armitage. Claire M. A. Haworth, Rui Adele Wang, Philip Collard and Oliver S. P. Davis read and approved the final manuscript.

## Supporting information

Supplementary Material

## Data Availability

The datasets analysed during the current study are not publicly available as the informed consent obtained from ALSPAC participants does not allow data to be made freely available through any third party maintained public repository. However, data used for this submission can be made available on request to the ALSPAC Executive. The ALSPAC data management plan describes in detail the policy regarding data sharing, which is through a system of managed open access. Full instructions for applying for data access can be found here: http://www.bristol.ac.uk/alspac/researchers/access/. The ALSPAC study website contains details of all the data that are available (http://www.bristol.ac.uk/alspac/researchers/our‐data/).
