# Supplementary material for: Positive wellbeing and resilience following adolescent victimisation: An exploration into protective factors across development
Source: JCPP Adv. 2021 Jul 15;1(2):e12024. doi: 10.1002/jcv2.12024 (PMC9386589; doi:10.1002/jcv2.12024)
Supplement: Supplementary file 1 — Supplementary Material [file JCV2-1-e12024-s001.docx]

**Positive wellbeing and resilience following adolescent victimisation: An exploration into protective factors across development**

Jessica M. Armitage^a^, R.Adele H. Wang^b^, Oliver S.P. Davis^c^, Philip Collard^a^ and Claire M.A. Haworth^a^

^a^ School of Psychological Science, University of Bristol.

^b^ School of Economics, Finance and Management, University of Bristol.

^c^ Population Health Sciences, Bristol Medical School.

**Contents**

**Supplementary methods**2

Power analyses2

Supplementary Tables and Figures3

**Figure S1:** Flowchart of participants in the Avon Longitudinal Study of Parents and Children3

**Table S1:** Response patterns across variables4

**Table S2:** Comparison of characteristics and mental health of different protective factor responders5

**Table S3:** Correlations between study variables6

**Table S4:** Correlations between study variables (complete dataset, n=949)8

**Table S5:** Variables included in multiple imputation10

**Table S6:** Impact of victimisation (untransformed), protective factors, and their interaction on wellbeing at 23 years11

**Table S7:** Impact of victimisation (untransformed), protective factors, and their interaction on life satisfaction and depressive symptoms at 23 years12

**Table S8:** Impact of victimisation (log-transformed and untransformed), social skills (log-transformed), and their interaction on wellbeing, life satisfaction and depressive symptoms aged 2323

**Table S9:** Loadings of principal components on the individual, family, and peer-level protective factors12

**Table S10:** Comparison of wellbeing scores among different protective factor responders in relation to experiences of victimisation13

**Table S11:** Impact of victimisation (log-transformed), protective factors, and their interaction on wellbeing at 23 years (full results)14

**Table S12:** Impact of victimisation (log-transformed), protective factors, and their interaction on wellbeing at 23 years (imputed dataset)16

**Table S13:** Impact of victimisation (log-transformed), protective factors, and their interaction on life satisfaction and depressive symptoms at 23 years17

**Table S14:** Impact of victimisation (log-transformed), principal components, and their interaction on wellbeing at 23 years21

**Table S15:** Impact of victimisation (log-transformed), principal components, and their interaction on life satisfaction and depressive symptoms22

**Supplementary methods**

**Power analyses**

Power calculations were conducted using previously reported regression coefficients and the formula outlined by Perugini et al., (2018):


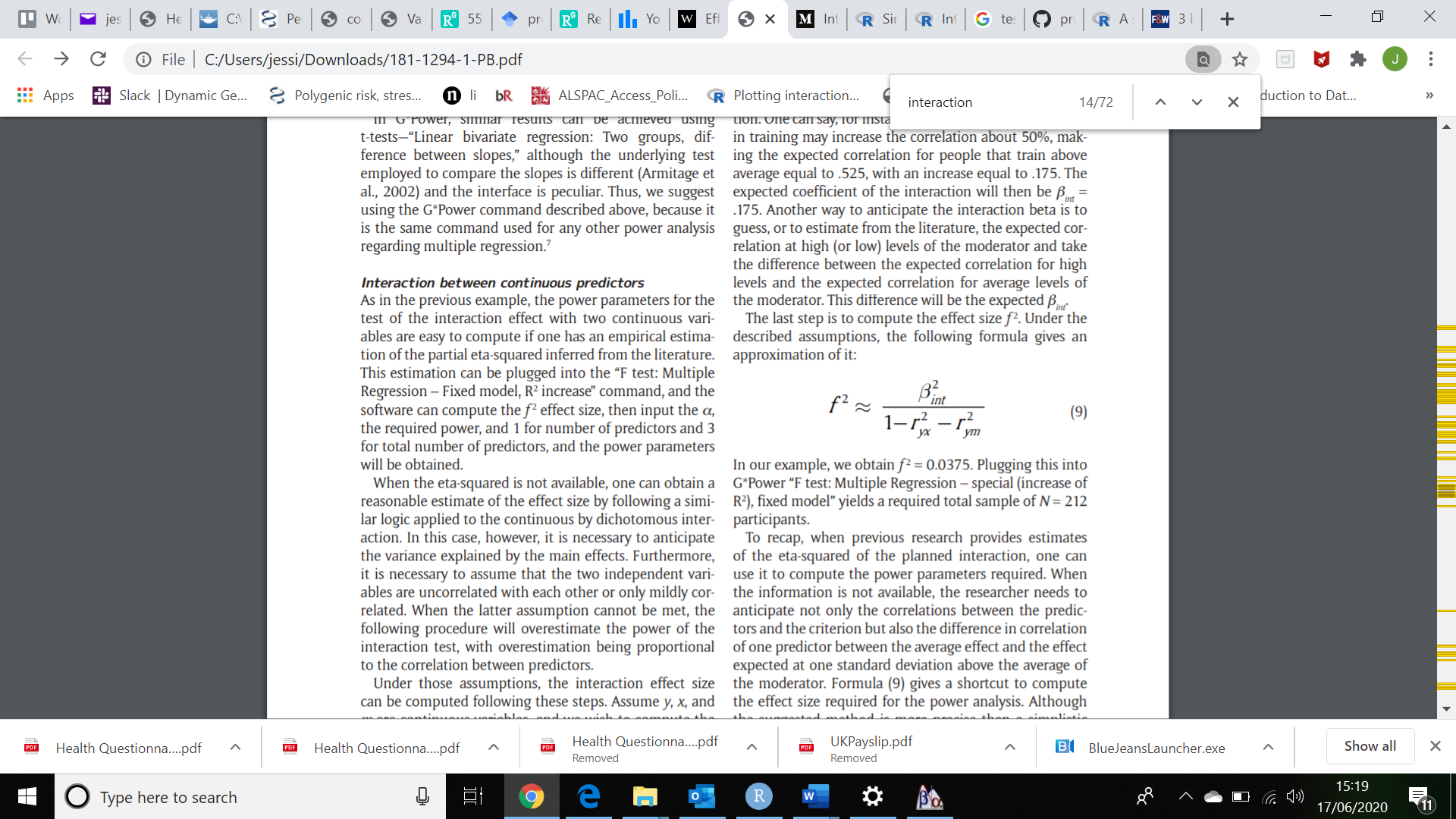


*ß*^2^*_int_* represents the interactive effect estimate, *r*^2^_yx_ represents the correlation between the outcome variable and predictor, and *r*^2^_ym_ represents the correlation between the outcome variable and the moderator. It has been recommended that if interaction effects are not available in the literature, researchers should estimate these using previously reported estimates of correlations between the study variables under the high or experimental condition (r_a_), and under the low or control condition (r_b_) (Perugini et al., 2018). Thus, to calculate the power needed to detect an interaction in predicting wellbeing, correlation estimates were taken from a study exploring the impact of peer support on life satisfaction among victims of bullying (Flaspohler et al., 2009). The interaction was estimated by subtracting the correlation between victimisation and life satisfaction among those with high peer support (r_a_=-0.27), from correlations among those with low peer support (r_b_=-0.19). Using the formula (below) resulted in *f*^2^ = 0.007320979. When plugged into G*Power version 3.1.9.7 (Faul et al., 2009), calculations revealed that a sample size of n=1494 would be necessary to achieve this estimate with 80% power. Analyses using the smallest sample in our study were conducted using n=1712 participants. All analyses therefore had above 80% power to detect these effects.

(^-^0.27 – ^-^0.19)^2^

1 – (^-^0.23^2^) – (^-^0.27)^2^


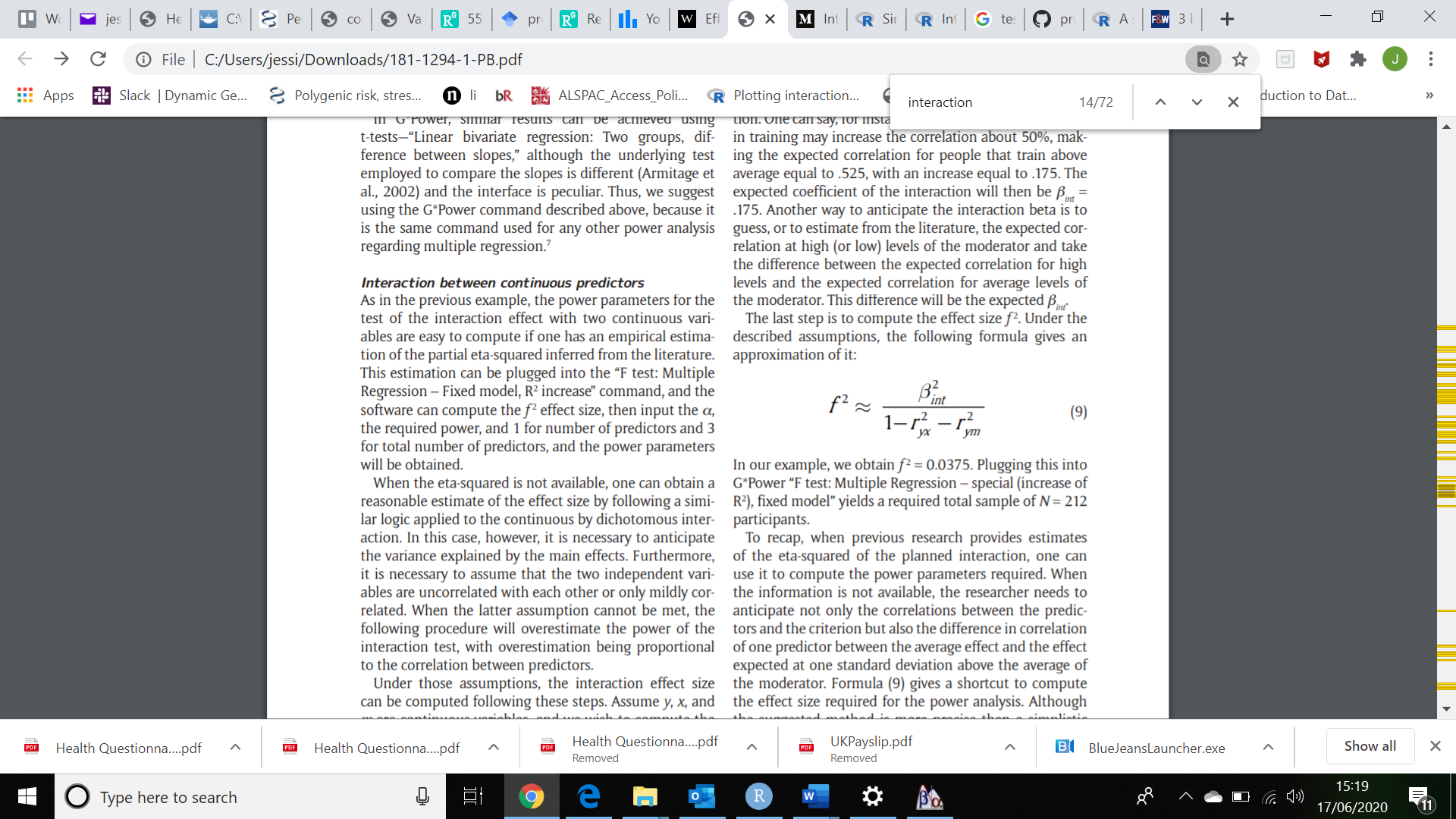


**Supplementary Tables and Figures**

Original enrolment phase (14541 eligible pregnancies)

Total ALSPAC sample as of 2019 (15454 eligible pregnancies)

Enrolment *phase III* (15247 eligible pregnancies)

Data also available on the following protective factors:

**Individual**

Scholastic competence (n=2302)

Global self-worth (n=2293)

Childhood social skills (n=2330)

Adolescent social skills (n=2339)

Late adolescent social skills (n=2092)

**Supplementary Figure S1:** Flowchart of participants in the Avon Longitudinal Study of Parents and Children

Data available on peer victimisation at 13 years (n=6532)

Data available on peer victimisation at 13 years and wellbeing at 23 years (n=3015)

Data available on peer victimisation, SES, and wellbeing (n=2703)

**Family**

Closeness to parents (n=1838)

Closeness to siblings (n=1712)

Family support (n=1833)

Parental involvement (n=1824)

Relationship with family (n=1838)

**Peer**

Childhood friendships (n=2303)

Adolescent friendships (n=2398)

Late adolescent friendships (n=1811)

Complete data on all protective factors, peer victimisation, SES, and wellbeing (n=941)

Data available on peer victimisation, SES, wellbeing and depressive symptoms (n=2624)

Data available on wellbeing at 23 years (n=4044)

| **Supplementary Table S1:** Response patterns across variables | | | | | | | |
| --- | --- | --- | --- | --- | --- | --- | --- |
|  | **Full sample in**  **ALSPAC (N)** | **% with SES data**^a^ | **% with victimisation data**^b^ | **% with wellbeing data**^c^ | **% with victimisation, SES, wellbeing**^d^ | **% with victimisation, SES, wellbeing, depression**^c^ | |
| **Predictor variable** |  |  |  |  |  |  | |
| Peer victimisation | 6529 | 5742 (87.9%) | 6529 (100%) | 2975 (45.6%) | 2624 (40.2%) | 2624 (40.2%) | |
| **Outcome variables** |  |  |  |  |  |  | |
| Wellbeing (WEMWBS) | 4044 | 3458 (85.6%) | 3015 (74.6%) | 4041 (100%) | 2624 (64.9%) | 2624 (64.9%) | |
| Life satisfaction | 4069 | 3486 (85.7%) | 3030 (74.5%) | 3993 (98.1%) | 2671 (65.6%) | 2597 (63.8%) | |
| Depressive symptoms | 3977 | 2669 (67.1%) | 2975 (74.8%) | 2929 (73.6%) | 2624 (66.0%) | 2624 (66.0%) | |
| **Protective factors** |  |  |  |  |  |  | |
| ***Individual-level*** |  |  |  |  |  |  | |
| Scholastic competence | 6854 | 6079 (89.0%) | 5292 (77.2%) | 2916 (42.5%) | 2367 (34.5%) | 2303 (33.6%) | |
| Global self-worth | 6843 | 6066 (88.6%) | 5283 (77.2%) | 2916 (42.6%) | 2362 (35.5%) | 2296 (33.6%) | |
| Childhood social skills | 7818 | 7263 (92.9%) | 5154 (65.9%) | 3072 (39.3%) | 2400 (30.7%) | 2330 (29.8%) | |
| Adolescent social skills | 6847 | 6169 (90.1%) | 5265 (76.9%) | 3110 (45.4%) | 2833 (41.4%) | 2339 (34.2%) | |
| Late adolescent social skills | 5413 | 4924 (91.0%) | 4285 (79.2%) | 2758 (51.0%) | 2516 (46.5%) | 2092 (38.6%) | |
| Academic ability | 6678 | 5937 (88.9%) | 5112 (76.5%) | 3236 (48.6%) | 2904 (43.5%) | 2360 (35.3%) | |
| ***Family-level*** |  |  |  |  |  |  | |
| Closeness to parents | 4030 | 3539 (87.8%) | 3431 (85.1%) | 2338 (58.0%) | 2088 (51.8%) | 1838 (45.6%) | |
| Closeness to siblings | 3766 | 3312 (87.9%) | 3198 (84.9%) | 2186 (58.0%) | 1954 (51.9%) | 1712 (45.6%) | |
| Family support | 4025 | 3528 (87.6%) | 3421 (85.0%) | 2337 (58.1%) | 2084 (51.8%) | 1833 (45.5%) | |
| Parental involvement | 3982 | 3498 (86.9%) | 3390 (85.1%) | 2321 (58.3%) | 2074 (52.1%) | 1824 (45.8%) | |
| Relationship with family | 4033 | 3539 (87.8%) | 3429 (85.0%) | 2340 (58.0%) | 2089 (51.8%) | 1838 (45.6%) | |
| ***Peer-level*** |  |  |  |  |  |  | |
| Childhood friendships | 6906 | 6119 (88.6%) | 5341 (77.3%) | 2926 (42.4%) | 2660 (38.5%) | 2303 (33.3%) | |
| Adolescent friendships | 6157 | 5405 (87.8%) | 6004 (97.5%) | 2800 (45.5%) | 2511 (40.8%) | 2398 (38.9%) | |
| Late adolescent friendships | 3963 | 3485 (87.9%) | 3375 (85.2%) | 2299 (58.0%) | 2058 (51.9%) | 1811 (45.7%) | |
| ^a^  Individuals with complete data on the measured variable, who were also assessed for SES.  ^b^ Individuals with complete data on the measured variable, who were also assessed for victimisation at 13 years.  ^c^ Individuals with complete data on the measured variable, who were also assessed for wellbeing at 23 years.  ^d^ Individuals with complete data on the measured variable, who were also assessed for victimisation aged 13 and wellbeing at 23 years. | | | | | | |  |

| **Table S2:** Comparison of characteristics and mental health of different protective factor responders | | | | | | | | | | | |
| --- | --- | --- | --- | --- | --- | --- | --- | --- | --- | --- | --- |
|  |  | | **Protective factor score** | **Sex** | **Ethnicity** | **Victimisation scores** | | **Parental social class^a^** | **Mental**  **wellbeing** | **Depressive symptoms** | |
| **Protective Factor** | | **N** | **Mean (SD)** | **(% female)** | **(% white)** | **Mean (SD)** | **Frequently victimised (%) ^b^** | **Mean (SD)** | **Mean (SD)** | **Mean (SD)** | **% with clinically relevant symptoms ^c^** |
| Scholastic competence | | 2302 | 17.28 (3.54) | 63.9 | 96.6 | 1.77 (2.61) | 17.0 | 5.29 (1.83) | 49.10 (8.91) | 6.67 (5.87) | 16.1 |
| Global self-worth | | 2296 | 19.40 (3.28) | 63.9 | 96.6 | 1.77 (2.61) | 17.1 | 5.30 (1.83) | 49.12 (8.91) | 6.66 (5.87) | 16.0 |
| Childhood social skills | | 2330 | 21.63 (3.14) | 63.1 | 96.7 | 1.76 (2.63) | 16.9 | 5.28 (1.80) | 49.21 (8.86) | 6.63 (5.79) | 16.0 |
| Adolescent social skills | | 2339 | 21.82 (3.25) | 62.4 | 96.8 | 1.73 (2.58) | 16.6 | 5.29 (1.82) | 49.20 (8.92) | 6.54 (5.78) | 15.1 |
| Late adolescent social skills | | 2092 | 21.44 (3.61) | 62.8 | 96.7 | 1.75 (2.62) | 16.7 | 5.24 (1.81) | 49.22 (8.87) | 6.54 (5.74) | 15.1 |
| Academic ability | | 2360 | 3.83 (0.52) | 64.5 | 96.7 | 1.78 (2.62) | 17.1 | 5.31 (1.83) | 49.12 (8.93) | 6.62 (5.4) | 15.7 |
| Closeness to parents | | 1838 | 2.38 (0.67) | 63.2 | 96.4 | 1.73 (2.57) | 16.3 | 5.25 (1.82) | 49.51 (8.60) | 6.52 (5.67) | 14.6 |
| Closeness to siblings | | 1712 | 2.20 (0.77) | 63.5 | 96.9 | 1.70 (2.53) | 16.1 | 5.24 (1.83) | 49.49 (8.60) | 6.50 (5.66) | 14.7 |
| Family support | | 1833 | 2.40 (1.13) | 63.2 | 96.4 | 1.73 (2.56) | 16.2 | 5.25 (1.82) | 49.50 (8.59) | 6.52 (5.68) | 14.7 |
| Parental involvement | | 1824 | 1.86 (1.04) | 63.4 | 96.5 | 1.72 (2.53) | 16.2 | 5.25 (1.83) | 49.51 (8.60) | 6.50 (5.67) | 14.6 |
| Relationship with family | | 1838 | 2.89 (0.94) | 63.3 | 96.4 | 1.74 (2.57) | 16.3 | 5.25 (1.82) | 49.51 (8.60) | 6.51 (5.67) | 14.6 |
| Childhood friendships | | 2303 | 11.63 (2.34) | 64.2 | 96.4 | 1.76 (2.59) | 16.8 | 5.29 (1.82) | 49.12 (8.91) | 6.68 (5.92) | 16.4 |
| Adolescent friendships | | 2398 | 12.07 (1.96) | 64.1 | 96.5 | 1.69 (2.48) | 16.5 | 5.36 (1.85) | 49.20 (8.91) | 6.60 (5.81) | 15.6 |
| Late adolescent friendships | | 1811 | 11.88 (2.40) | 64.0 | 96.4 | 1.69 (2.53) | 16.4 | 5.26 (1.82) | 49.66 (8.58) | 6.51 (5.69) | 14.7 |
| *Note:*  N represents the number of participants who had complete data on the protective factor measure, the victimisation scale, the depression and wellbeing assessments, and SES.  **^a^** Parental social class is based on mother and partner responses to the education and occupational status items at 18 weeks gestation. Overall scores range from 2 to 11, with higher scores representing lower SES.  **^b^** Individuals were classified as ‘frequently victimised’ if they scored above 4 on the Bullying and Friendship Interview Schedule.  **^c^** Clinically relevant symptoms based on those scoring 12 or more on the Moods and Feelings Questionnaire. | | | | | | | | | | | |

| **Table S3:** Correlations between study variables | | | | | | | | | | | | | | | | | | | | |
| --- | --- | --- | --- | --- | --- | --- | --- | --- | --- | --- | --- | --- | --- | --- | --- | --- | --- | --- | --- | --- |
| **Correlation matrix** | | | | | | | | | | | | | | | | | | | | |
| Variables | 1 | 2 | 3 | 4 | 5 | 6 | 7 | 8 | 9 | 10 | 11 | 12 | 13 | 14 | 15 | 16 | 17 | 18 | 19 |  |
| 1. Peer victimisation (log) | 1 | **-.12*****  (-.16,  -.09) | .**17*****  (.14, .21) | **-.13*****  (-.17,  -.10) | -.02  (-.05, .003 | **-.08*****  (-.11,  -.05) | **-.09*****  (-.12,  -.06) | **-.12*****  (-.15,  -.09) | **-.12*****  (-.15,  -.09) | **-.14*****  (-.17,  -.11) | **-.03***  (-.06,  -.005) | **-.05*****  (-.09,  -.02) | **-.06*****  (-.09,  -.03) | **-.07*****  (-.11,  -.03) | **-.04****  (-.08,  -.01) | **-.12*****  (-.15,  -.08) | **-.10*****  (-.13,  -.08) | **-.19*****  (-.21,  -.16) | **-.10*****  (-.13,  -.06) |  |
| 2. Mental wellbeing |  | 1 | **-.69*****  (-.71,  -.67) | **.66*****  (.64, .68) | **-.07*****  (-.10,  -.04) | **.09*****  (.06, .13) | **.11*****  (.08, .15) | **.10*****  (.06, .13) | **.11*****  (.08, .15) | **.13*****  (.10, .17) | **.16*****  (.13, .20) | **.14*****  (.10, .18) | **.14*****  (.10, .18) | .**18*****  (.14, .22) | **.13*****  (.09, .17) | **.18*****  (.14, .21) | **.08*****  (.04, .11) | **.11*****  (.08, .15) | **.21*****  (.17, .25) |  |
| 3. Depressive symptoms |  |  | 1 | **-.58*****  (-.60  -.56) | **.07*****  (.04, .10) | **-.10*****  (-.14,  -07) | **-.10*****  (-.14,  -07) | **-.08*****  (-.12,  -.04) | **-.13*****  (-.16,  -.09) | **-.16*****  (-.19,  -.12) | **-.14*****  (-.17,  -.10) | **-.14*****  (-.18,  -.10) | **-.11*****  (-.15,  -.07) | **-.16*****  (-.19,  -.12) | **-.15*****  (-.19,  -.11) | **-.18*****  (-.22,  -.14) | **-.08*****  (-.12,  -.05) | **-.11*****  (-.15,  -.07) | **-.15*****  (-.20,  -.11) |  |
| 4. Life satisfaction |  |  |  | .1 | **-.06*****  (-.09,  -.02) | **.09*****  (.06, .13) | **.11*****  (.08, .15) | **.12*****  (.09, .16) | **.16*****  (.13, .19) | **.15*****  (.12, .19) | **.14*****  (.10, .17) | **.15*****  (.11, .19) | **.12*****  (.07, .16) | .**18*****  (.14, .21) | **.14*****  (.10, .18) | **.18*****  (.14, .21) | **.08*****  (.04, .12) | **.13*****  (.09, .16) | **.19*****  (.15, .23) |  |
| 5. Low SES |  |  |  |  | 1 | **-.10*****  (-.13,  -.08) | **-.03*****  (-.06,  -.01) | **-.06*****  (-.08,  -.04) | **-.04*****  (-.07,  -.02) | **-.04***  (-.06,  -.01) | **-.12*****  (-.15,  -.10) | -.03  (-.06,  .001) | **-.07*****  (-.11,  -.04) | -.001  (-.03,  .03) | **-.20*****  (-.24,  -.17) | **-.05*****  (-.08,  -.02) | -.002  (-.03,  .02) | **.06*****  (.04,  .09) | .03  (-.008, .06) |  |
| 6. Scholastic competence |  |  |  |  |  | 1 | **.40*****  (.38, .42) | **.06*****  (.03, .09) | .02  (-.01, .04) | **.03***  (.01, .06) | **.23*****  (.20, .26) | .03  (-.005, .06) | **.05*****  (.01, .08) | **.07*****  (.03, .10) | **.06*****  (.03, .10) | **.05*****  (.02, .09) | **.17*****  (.14, .19) | **.08*****  (.05, .11) | .02  (-.02, .05) |  |
| 7. Global self-worth |  |  |  |  |  |  | 1 | **.12*****  (0.10, 0.15) | **.06*****  (.03, .08) | **.07*****  (.04, .10) | **.11*****  (.08, .14) | **.08*****  (.04, .11) | **.06*****  (.03, .10) | **.08*****  (.05, .12) | **.06*****  (.03, .10) | .**07*****  (.03, .10) | **.20*****  (.18, .23) | **.11*****  (.08, .14) | **.07*****  (.04, .11) |  |
| 8. Childhood social skills |  |  |  |  |  |  |  | 1 | **.54*****  (.52, .56) | **.43*****  (.41, .45) | **.08*****  (.06, .11) | **.12*****  (.08, .15) | **.11*****  (.07, .14) | **.06*****  (.03, .10) | **.06*****  (.02, .09) | **.10*****  (.07, .14) | .**09*****  (.06, .11) | **.05*****  (.02, .08) | .03  (-.02, .07) |  |
| 9. Adolescent social skills |  |  |  |  |  |  |  |  | 1 | **.61*****  (.59, .63) | **.12*****  (.09, .15) | **.14*****  (.11, .17) | **.09*****  (.05, .12) | **.07*****  (.04, .11) | **.06*****  (.02, .09) | **.15*****  (.11, .18) | **.04*****  (.02, .07) | **.03***  (.004, .06) | .02  (-.01, .06) |  |
| 10. Late adolescent social skills |  |  |  |  |  |  |  |  |  | 1 | **.07*****  (.04, .10) | **.19*****  (.16, .23) | **.12*****  (.08, .15) | **.12*****  (.08, .15) | **.11*****  (.07, .14) | **.21*****  (.17, .24) | **.04*****  (.01, .07) | .03  (-.03, .06) | .01  (-.03, .05) |  |
| 11. Self-perceived academic ability |  |  |  |  |  |  |  |  |  |  | 1 | **.08*****  (.05, .12) | **.06*****  (.02, .10) | **.09*****  (.05, .12) | **.11*****  (.08, .14) | **.09*****  (.05, .12) | **.09*****  (.06, .11) | **.08*****  (.06, .11) | **.06*****  (.02, .09) |  |
| 12. Parental closeness |  |  |  |  |  |  |  |  |  |  |  | 1 | **.45*****  (.42, .48) | **.53*****  (.50, .55) | **.27*****  (.24, .30) | **.61*****  (.59, .63) | **.07*****  (.03, .10) | **.07*****  (.04, .11) | **.18*****  (.14, .21) |  |
| 13. Sibling closeness |  |  |  |  |  |  |  |  |  |  |  |  | 1 | **.41*****  (.38, .43) | **.22*****  (.19, .25) | **.41*****  (.38, .44) | **.07*****  (.04, .11) | **.10*****  (.06, .14) | **.15*****  (.12, .18) |  |
| 14. Family support |  |  |  |  |  |  |  |  |  |  |  |  |  | 1 | **.29*****  (.26, .32) | **.54*****  (.51, .56) | **.09*****  (.06, .13) | **.10*****  (.07, .14) | **.22*****  (.19, .25) |  |
| 15. Family involvement |  |  |  |  |  |  |  |  |  |  |  |  |  |  | 1 | **.38*****  (.35, .40) | .03  (-.006, .06) | -.03  (-.07, .04) | .02  (-.02, .05) |  |
| 16. Family cohesion |  |  |  |  |  |  |  |  |  |  |  |  |  |  |  | 1 | **.06*****  (.03, .10) | **.07*****  (.04, .11) | **.14*****  (0.11, 0.17) |  |
| 17. Childhood friendships |  |  |  |  |  |  |  |  |  |  |  |  |  |  |  |  | 1 | **.18*****  (.15, .21) | **.14*****  (.10, .17) |  |
| 18. Adolescent friendships |  |  |  |  |  |  |  |  |  |  |  |  |  |  |  |  |  | 1 | **.23*****  (.19, .26) |  |
| 19. Late adolescent friendships |  |  |  |  |  |  |  |  |  |  |  |  |  |  |  |  |  |  | 1 |  |
| *Note:*  Variables 6-11 comprise of protective factors at the individual level, variables 12-17 comprise of family-level protective factors, and 18-19 are the peer-level protective factors. Correlations were conducted using samples with available information related to the included measures only, therefore samples ranged from n=2185 to n=7261.  ***p<0.001, **p<0.01, *p<0.05. | | | | | | | | | | | | | | | | | | | | |

| **Table S4:** Correlations between study variables (complete dataset, n=949) | | | | | | | | | | | | | | | | | | | | |
| --- | --- | --- | --- | --- | --- | --- | --- | --- | --- | --- | --- | --- | --- | --- | --- | --- | --- | --- | --- | --- |
| **Correlation matrix** | | | | | | | | | | | | | | | | | | | | |
| Variables | 1 | 2 | 3 | 4 | 5 | 6 | 7 | 8 | 9 | 10 | 11 | 12 | 13 | 14 | 15 | 16 | 17 | 18 | 19 |  |
| 1. Peer victimisation (log) | 1 | -.06  (-.12,  .002) | **.12*****  (.06, .19) | **-.06***  (-.13,  .005) | -.002  (-.07, .06) | -.06  (-.12,  .007) | **-.09***  (-.15,  -.02) | **-.13*****  (-.20,  -.07) | **-.14*****  (-.21,  -.08) | **-.13*****  (-.20,  -.07) | -.04  (-.10,  .03) | -.04  (-.10,  .03) | -.05  (-.12,  .01) | -.04  (-.10,  .03) | .03  (-.03,  .10) | **-.11*****  (-.17,  -.04) | **-.10*****  (-.16,  -.04) | **-.19*****  (-.26,  -.13) | **-.10*****  (-.17,  -.04) |  |
| 2. Wellbeing |  | 1 | **-.70*****  (-.73,  -.66) | **.63*****  (.59,  .67) | -.05  (-.12,  .01) | **.07***  (.008, .14) | **.10*****  (.04, .16) | **.10*****  (.04, .16) | **.08****  (.03, .16) | **.12*****  (.06, .18) | **.12*****  (.06, .18) | **.16*****  (.10, .23) | **.13*****  (.06, .19) | **.18*****  (.11, .24) | **.12*****  (.05, .18) | **.20*****  (.14, .26) | .05  -.02, .11) | .05  (-.009, .12) | **.24*****  (.18, .30) |  |
| 3. Depressive symptoms |  |  | 1 | **-.58*****  (-.62,  -.53) | .05  (-.02, 0.11) | -.06  (-.12,  .004) | **-.13*****  (-.19,  -07) | **-.07***  (-.14,  -.009) | **-.11*****  (-.18,  -.05) | **-.11*****  (-.18,  -.05) | **-.12*****  (-.18,  -.06) | **-.11*****  (-.18,  -.05) | **-.11*****  (-.17,  -.04) | **-.13*****  (-.19,  -.06) | **-.09*****  (-.16,  -.03) | **-.14*****  (-.20,  -.07) | -.04  (-.10,  .03) | -.05  (-.12,  .01) | **-.16*****  (-.22,  -.10) |  |
| 4. Life Satisfaction |  |  |  | 1 | -.05  (-.11,  .01) | .04  (-.03, .10) | **.09*****  (.03, .16) | **.16*****  (.10, .23) | **.15****  (.08, .21) | **.14*****  (.08, .21) | **.07****  (.007, .14) | **.15*****  (.08, .21) | **.11*****  (.05, .18) | **.15*****  (.09, .21) | **.12*****  (.06, .19) | **.17*****  (.11, .24) | .03  -.03, .10) | .04  (-.02, .11) | **.20*****  (.14, .26) |  |
| 5. Low SES |  |  |  |  | 1 | **-.13*****  (-.19,  -.07) | .02  (-.04,  -.09) | **-.10*****  (-.17,  -.04) | -.008  (-.07,  .06) | -.01  (-.08,  .05) | **-.13*****  (-.20,  -.07) | -.01  (-.08,  .05) | **-.08***  (-.14,  -.01) | -.01  (-.08,  .05) | **-.18*****  (-.25,  -.12) | -.07  (-.14,  -.007) | -.03  (-.10,  .03) | .01  (-.05,  .08) | -.03  (-.10, .03) |  |
| 6. Scholastic competence |  |  |  |  |  | 1 | **.40*****  (.34, .45) | **.07***  (.009, .14) | .02  (-.05, .08) | .005  (-.06, .07) | **.23*****  (.16, .29) | .03  (-.04, .09) | .06  (-.008, .12) | .06  (-.005, .12) | .06  (-.006, .12) | .04  (-.03, .10) | **.17*****  (.11,  .23) | **.09*****  (.03, .16) | .06  (-.10, .12) |  |
| 7. Global self-esteem |  |  |  |  |  |  | 1 | **.13*****  (.07, .20) | .03  (-.04, .10) | **.07***  (.009, .14) | **.15*****  (.08, .21) | **.08***  (.01, .14) | .06  (-.004, .12) | **.09***  (.02, .15) | .02  (-.04, .09) | .06  (-.009, .12) | **.21*****  (.15, .27) | **.10*****  (.04, .16) | **.13*****  (.06, .19) |  |
| 8. Childhood social skills |  |  |  |  |  |  |  | 1 | **.56*****  (.52, .61) | **.40*****  (.34, .45) | **.07***  (.005, .13) | **.11*****  (.04, .17) | **.07***  (.002, .13) | .06  (-.007, .12) | .03  (-.03, .10) | **.11*****  (.05, .17) | **.12*****  (.06, .19) | .05  (-.01, .12) | **.09*****  (.03, .16) |  |
| 9. Adolescent social skills |  |  |  |  |  |  |  |  | 1 | **.58*****  (.53, 0.62) | **.10*****  (.03, .16) | .**13*****  (.06, .19) | .**08***  (.02, .15) | .06  (-.003, .13) | .03  (-.04, .09) | **.13*****  (.07, .20) | **.07***  (.009, .14) | .02  (-.05, .08) | .04  (-.02, .10) |  |
| 10. Late adolescent social skills |  |  |  |  |  |  |  |  |  | 1 | .05  (-.02, .11) | **.17*****  (.11, .23) | **.08***  (.02, .15) | **.08***  (.01, .14) | .03  (-.04, .09) | **.14*****  (.08, .21) | **.07***  (.009, .14) | -.02  (-.08, .05) | .03  (-.04, .09) |  |
| 11. Academic ability |  |  |  |  |  |  |  |  |  |  | 1 | .05  (-.02, .11) | .05  (-.02, .11) | .02  (-.04, .09) | **.16*****  (.10, .22) | **.08***  (.01, .14) | **.12*****  (.06, .19) | **.09***  (.03, .15) | **.09***  (.03, .16) |  |
| 12. Parental closeness t |  |  |  |  |  |  |  |  |  |  |  | 1 | **.43*****  (.38, .48) | **.49*****  (.44, .54) | **.24*****  (.17, .30) | **.59*****  (.54, .63) | .04  (-.03, .10) | **.09*****  (.02, .15) | **.18*****  (.12, .24) |  |
| 13. Sibling closeness |  |  |  |  |  |  |  |  |  |  |  |  | 1 | **.39*****  (.34, .44) | **.22*****  (.16, .28) | **.41*****  (.35, .46) | .03  (-.03, .09) | **.15*****  (.08, .21) | **.18*****  (.12, .25) |  |
| 14. Family support |  |  |  |  |  |  |  |  |  |  |  |  |  | 1 | **.25*****  (.19, .31) | **.52*****  (.47, .57) | .03  (-.04, .09) | **.10*****  (.04, .17) | **.20*****  (.13, .26) |  |
| 15. Family involvement |  |  |  |  |  |  |  |  |  |  |  |  |  |  | 1 | **.33*****  (.27, .39) | .03  (-.04, .10) | .01  (-.08, .05) | .007  (-.006, .07) |  |
| 16. Family cohesion |  |  |  |  |  |  |  |  |  |  |  |  |  |  |  | 1 | -.01  (-.07, .05) | .06  (-.003, .13) | **.17*****  (.10, .23) |  |
| 17. Childhood friendships |  |  |  |  |  |  |  |  |  |  |  |  |  |  |  |  | 1 | **.17*****  (.11, .23) | **.09*****  (.03, .16) |  |
| 18. Adolescent friendships |  |  |  |  |  |  |  |  |  |  |  |  |  |  |  |  |  | 1 | **.26*****  (.20, .32) |  |
| 19. Late adolescent friendships |  |  |  |  |  |  |  |  |  |  |  |  |  |  |  |  |  |  | 1 |  |
| *Note:*  Variables 6-11 comprise of protective factors at the individual level, variables 12-17 comprise of family-level protective factors, and 18-19 are the peer-level protective factors. Correlations were conducted using samples with available information related to all measures, therefore the sample was restricted to n=949.  ***p<0.001, **p<0.01, *p<0.05. | | | | | | | | | | | | | | | | | | | | |

| **Table S5:** Variables included in multiple imputation | | | | | | | |  |
| --- | --- | --- | --- | --- | --- | --- | --- | --- |
| **Variable** | **Measure** | **Age at assessment** | **Question answered by** | **Number of items** | **Sample item** | **Item response options** | **Item scoring** | |
| Ethnicity of child | 1 item | 32 weeks gestation | Mother | 2 | “How would you describe the race or ethnic group of yourself/your partner?” | 8 response options, including ‘Other (please describe)’. | Child’s ethnic background defined as non-white if either mother or father are non-white | |
| Mother’s age at first pregnancy | 1 item | 18 weeks gestation | Mother | 1 | “How old were you when you became pregnant for the very first time?” | Open response. | Higher score signifies higher age | |
|  |  |  |  |  |  |  |  | |
| Homeownership status | 1 item | 8 months | Mother | 1 | “Do you currently live in..” | 6 response options including “Mortgaged” and “Rented from private landlord” | Higher score signifies greater home ownership | |
| Mother marital status | 1 item | 32 weeks gestation | Mother | 1 | “What is your present marital status? | 6 response options including “Married” and “Separated” | High score signifies currently married | |
|  |  |  |  |  |  |  |  | |
| Mother and partner educational qualifications | 2 items | 32 weeks gestation | Mother | 2 | “What educational qualifications do you/your partner have?” | List of qualifications, respondent must tick all that apply | Higher score signifies more educational qualifications | |
| Maternal smoking during pregnancy | 1 item | 32 weeks gestation | Mother | 1 | “How many cigarettes per day are you yourself smoking?” | Open response | Greater tar intake | |
| Maternal depression | Edinburgh Postnatal Depression Scale^a^ | 32 weeks gestation | Mother | 10 | “Felt sad/miserable in past week” | 4-point scale ranging, including ‘Yes, most of the time” | Greater depressive symptoms | |
|  |  |  |  |  |  |  |  | |
| Adolescent depressive symptoms | Moods and Feelings Questionnaire^b^ | 13 | Child | 9 | “Teenager felt miserable or unhappy” | 3-pont scale ranging from “Not at all” to “True” | Greater depressive symptoms | |
| Childhood IQ | Wechsler Intelligence Scale for Children (WISC)^c^ | 8 | Child | - | - | Responses range from 45 to 151. | Higher IQ | |
| *Note:*  Multiple imputation was conducted using these sociodemographic factors. These were selected as they have previously been associated with missingness in ALSPAC^d^. In total, 60 imputations were ran using Chained Equations (MICE) in R.  ^a^ Cox, J. L., Holden, J. M., & Sagovsky, R. (1987). Detection of postnatal depression: Development of the 10-item Edinburgh Postnatal Depression Scale. *British Journal of Psychiatry, 150,* 782-786.  ^b^ Angold, A., Costello, E. J., Messer, S. C., Pickles, A., Winder, F., & Silver, D. (1995). The development of a short questionnaire for use in epidemiological studies of depression in children and adolescents. *International Journal of Methods in Psychiatric Research*, 5, 237-249.  ^c^ Wechsler, D. (1949). Wechsler Intelligence Scale for Children. San Antonio, TX, US: Psychological Corporation  ^d^ Houtepen, L. C., Heron, J., Suderman, M. J., Tilling, K., & Howe, L. D. (2018). Adverse childhood experiences in the children of the Avon Longitudinal Study of Parents and Children (ALSPAC). Wellcome open research, 3, 106. https://doi.org/10.12688/wellcomeopenres.14716.1. | | | | | | | | |

| **Table S6:** Impact of victimisation (untransformed), protective factors, and their interaction on wellbeing at 23 years | | | | | | | | | | | | | | | | | | | | | | | | | | | | | | |
| --- | --- | --- | --- | --- | --- | --- | --- | --- | --- | --- | --- | --- | --- | --- | --- | --- | --- | --- | --- | --- | --- | --- | --- | --- | --- | --- | --- | --- | --- | --- |
|  |  | |  | | | | **Wellbeing** | | | | | | | | | | | | | | | | | | | |  | |  |  |
|  | |  | | | **Protective Factor** | | | | | | |  | | | | **Victimisation** | | |  | | | **Interaction** | | | | | | | | |
|  | |  | | | **N** | | | **β (95% C.I.)** | **SE** | **P value** |  | | | | **β (95% C.I)** | | **SE** | **P value** | |  | | **β (95% C.I)** | **SE** | **P value** | | **R^2^** | | **ΔR^2^** | | |
| **Individual-level** | | | |  | |  | |  |  |  | | |  |  | | |  |  | |  | |  |  | |  |  | |  | | |
| Scholastic competence | | | |  | | 2302 | | .223 (-.233, .678) | .232 | .337 | | |  | -.380 (-.519, -.241) | | | .071 | **9.5x10^-8^** | |  | .170 (.038, .302) | | .067 | **.011** | | 2.63% | | .54% | | |
| Global self-worth | | | |  | | 2296 | | .774 (.310, 1.24) | .237 | **.001** | | |  | -.379 (-.519, -.238) | | | .072 | **1.3x10^-7^** | |  | .048 (-.086, .181) | | .068 | .483 | | 2.97% | | .81% | | |
| Childhood social skills | | | |  | | 2330 | | 1.29 (.775, 1.80) | .261 | **8.9x10^-7^** | | |  | -.382 (-.519, -.244) | | | .070 | **5.5x10^-8^** | |  | -.081 (-.203, .041) | | .062 | .191 | | 3.15% | | 1.02% | | |
| Adolescent social skills | | | |  | | 2339 | | 1.29 (.796, 1.78) | .250 | **2.9x10^-7^** | | |  | -.379 (-.518, -.240) | | | .070 | **9.6x10^-8^** | |  | -.005 (-.132, .121) | | .065 | .934 | | 3.61% | | 1.53% | | |
| Late adolescent social skills | | | |  | | 2092 | | 1.29 (.803, 1.79) | .251 | **2.7x10^-7^** | | |  | -.336 (-.480, -.193) | | | .073 | **4.7x10^-6^** | |  | .013 (-.113, .139) | | .064 | .836 | | 3.82% | | 1.91% | | |
| Academic ability | | | |  | | 2360 | | 1.32 (.876, 1.76) | .225 | **3.2x10^-7^** | | |  | -.382 (-.518, -.247) | | | .069 | **1.4x10^-7^** | |  | .049 (-.080, .178) | | .066 | .461 | | 4.51% | | 2.31% | | |
| **Family-level** | | | |  | |  | |  |  |  | | |  |  | | |  |  | |  |  | |  |  | |  | |  | | |
| Closeness to parents | | | |  | | 1838 | | 1.19 (.068, 1.69) | .258 | **4.5x10^-6^** | | |  | -.311 (-.463, -.159) | | | .077 | **6.3x10^-5^** | |  | .075 (-.072, .222) | | .075 | .318 | | 3.80% | | 2.11% | | |
| Closeness to siblings | | | |  | | 1712 | | 1.48 (.096, 1.99) | .262 | **2.1x10^-8^** | | |  | -.339 (-.497, -.181) | | | .081 | **2.7x10^-5^** | |  | -.009 (-.248, .070) | | .081 | .274 | | 3.76% | | 2.08% | | |
| Family support | | | |  | | 1833 | | 1.37 (.894, .185) | .243 | **2.1x10^-8^** | | |  | -.301 (-.455, -.147) | | | .078 | **1.2x10^-4^** | |  | .066 (-.076, .208) | | .072 | .359 | | 4.46% | | 2.74% | | |
| Family involvement | | | |  | | 1824 | | .767 (.278, 1.26) | .250 | **.002** | | |  | -.317 (-.471, -.163) | | | .078 | **5.7x10^-5^** | |  | .141 (-.009, .292) | | .077 | .067 | | 2.86% | | 1.40% | | |
| Family cohesion | | | |  | | 1838 | | 1.23 (.732, 1.73) | .255 | **1.5x10^-6^** | | |  | -.268 (-.214, -.115) | | | .078 | **6.0x10^-4^** | |  | .098 (-.050, .246) | | .075 | .194 | | 4.24% | | 2.53% | | |
| **Peer-level** | | | |  | |  | |  |  |  | | |  |  | | |  |  | |  |  | |  |  | |  | |  | | |
| Childhood friendships | | | |  | | 2303 | | .557 (.101, 1.01) | .232 | **.017** | | |  | -.401 (-.545, -.257) | | | .073 | **5.1x10^-8^** | |  | .004 (-.124, .132) | | .065 | .957 | | 2.67% | | .29% | | |
| Adolescent friendships | | | |  | | 2398 | | .974 (.523, 1.42) | .230 | **2.4x10^-5^** | | |  | -.382 (-.536, -.228) | | | .078 | **1.2x10^-6^** | |  | -.020 (-.140, .101) | | .061 | .749 | | 3.14% | | .95% | | |
| Late adolescent friendships | | | |  | | 1811 | | 2.29 (1.81, 2.76) | .245 | **2.0x10^-16^** | | |  | -.331 (-.490, -.172) | | | .081 | **4.6x10^-5^** | |  | -.204 (-.349, -.006) | | .074 | **.006** | | 6.86% | | 5.13% | | |
| *Note:*  All models adjusted for sex and socioeconomic status.  R^2^ is the variance accounted for by the main and interactive effects of victimisation and the protective factor, as well as the covariates. ΔR^2^ represents the incremental R^2^. This is the percentage of variance explained by the addition of the protective factor. The ΔR^2^ was calculated by regressing the outcome on victimisation and the covariates, and then including the interaction term with the protective factor and comparing the variance explained. | | | | | | | | | | | | | | | | | | | | | | | | | | | | | | |

| **Table S7:** Impact of victimisation (untransformed), protective factors, and their interaction on life satisfaction and depressive symptoms at 23 years | | | | | | | | | | | | | | | | | | | | | | | | | | | | | | | |
| --- | --- | --- | --- | --- | --- | --- | --- | --- | --- | --- | --- | --- | --- | --- | --- | --- | --- | --- | --- | --- | --- | --- | --- | --- | --- | --- | --- | --- | --- | --- | --- |
|  |  | |  | | | | **Impact on life satisfaction using untransformed victimisation scores** | | | | | | | | | | | | | | | | | | |  | |  | | |  |
|  | |  | | | **Protective Factor** | | | | | | |  | | | **Victimisation** | | |  | | | **Interaction** | | | | | | | | | |  |
|  | |  | | | **N** | | | **β (95% C.I.)** | **SE** | **P value** |  | | | **β (95% C.I)** | | **SE** | **P value** | |  | **β (95% C.I)** | | **SE** | **P value** | | **R^2^** | | | | **ΔR^2^** | | |
| **Individual-level** | | | |  | |  | |  |  |  | | |  |  | |  |  | |  |  | |  | |  |  | | | |  | | |
| Scholastic competence | | | |  | | 2310 | | .351 (.016, .687) | .171 | **.040** | | |  | -.320 (-.422, -.218) | | .052 | **9.0x10^-10^** | |  | .054 (-.041, .148) | | .048 | .264 | | 3.09% | | | | .71% | | |
| Global self-worth | | | |  | | 2304 | | .624 (.290, .967) | .175 | **3.7x10^-4^** | | |  | -.317 (-.420, .215) | | .052 | **1.6x10^-9^** | |  | .021 (-.077, .119) | | .050 | .677 | | 3.59% | | | | 1.14% | | |
| Childhood social skills | | | |  | | 2339 | | .821 (.505, 1.14) | .161 | **3.7x10^-7^** | | |  | -.292 (-.391, -.192) | | .051 | **1.1x10^-8^** | |  | -.167 (-.313, -.021) | | .075 | **.025** | | 3.46% | | | | 1.25% | | |
| Adolescent social skills | | | |  | | 2353 | | 1.04 (.679, 1.40) | .183 | **1.4x10^-8^** | | |  | -.291 (-.392, -.191) | | .051 | **1.4x10^-8^** | |  | .028 (-.065, .121) | | .047 | .553 | | 4.76% | | | | 2.39% | | |
| Late adolescent social skills | | | |  | | 2100 | | .989 (.627, 1.35) | .185 | **9.4x10^-8^** | | |  | -.275 (-.379, -.170) | | .053 | **2.7x10^-7^** | |  | .021 (-.071, .113) | | .047 | .659 | | 4.70% | | | | 2.36% | | |
| Academic ability | | | |  | | 2370 | | .997 (.671, 1.32) | .167 | **2.4x10^-9^** | | |  | -.314 (-.413, -.215) | | .050 | **5.4x10^-10^** | |  | -.030 (-.126, .066) | | .049 | .538 | | 4.39% | | | | 1.84% | | |
| **Family-level** | | | |  | |  | |  |  |  | | |  |  | |  |  | |  |  | |  |  | |  | | | |  | | |
| Closeness to parents | | | |  | | 1850 | | 1.05 (.672, 1.43) | .192 | **5.5x10^-8^** | | |  | -.287 (-.400, -.175) | | .057 | **6.3x10^-7^** | |  | -.020 (-.129, .090) | | .056 | .726 | | 4.22% | | | | 2.11% | | |
| Closeness to siblings | | | |  | | 1722 | | .844 (.457, 1.23) | .197 | **1.9x10^-5^** | | |  | -.328 (-.446, -.211) | | .060 | **4.9x10^-8^** | |  | -.050 (-.168, .069) | | .061 | .412 | | 3.17% | | | | 1.16% | | |
| Family support | | | |  | | 1845 | | .910 (.552, 1.27) | .183 | **6.7x10^-7^** | | |  | -.276 (-.389, -.162) | | .058 | **2.2x10^-6^** | |  | .062 (-.043, .168) | | .054 | .244 | | 4.46% | | | | 2.32% | | |
| Family involvement | | | |  | | 1836 | | .732 (.368, 1.10) | .186 | **8.3x10^-5^** | | |  | -.291 (-.404, -.177) | | .058 | **5.9x10^-7^** | |  | .066 (-.044, .177) | | .056 | .240 | | 3.56% | | | | 1.61% | | |
| Family cohesion | | | |  | | 1850 | | 1.02 (.647, 1.40) | .192 | **1.1x10^-7^** | | |  | -.257 (-.371, .143) | | .058 | **1.1x10^-5^** | |  | .022 (-.088, .133) | | .056 | .694 | | 4.56% | | | | 2.41% | | |
| **Peer-level** | | | |  | |  | |  |  |  | | |  |  | |  |  | |  |  | |  |  | |  | | | |  | | |
| Childhood friendships | | | |  | | 2313 | | .446 (.107, .784) | .173 | **.009** | | |  | -.352 (-.459, -.246) | | .054 | **9.0x10^-11^** | |  | -.066 (-.160, .028) | | .048 | .171 | | 2.93% | | | | .20% | | |
| Adolescent friendships | | | |  | | 2408 | | .630 (.297, .963) | .170 | **2.0x10^-4^** | | |  | -.306 (-.418, -.194) | | .057 | **9.8x10^-8^** | |  | -.017 (-.105, .070) | | .045 | .701 | | 3.13% | | | | .68% | | |
| Late adolescent friendships | | | |  | | 1823 | | 1.38 (1.02, 1.73) | .181 | **4.7x10^-14^** | | |  | -.292 (-.407, -.178) | | .058 | **6.3x10^-7^** | |  | -.096 (-.198, .007) | | .052 | .068 | | 5.51% | | | | 3.36% | | |
|  |  | |  | | | | **Impact on depressive symptoms using untransformed victimisation scores** | | | | | | | | | | | | | | | | | |  | | | |  | | |
| **Individual-level** | | | |  | |  | |  |  |  | | |  |  | |  |  | |  |  | |  |  | |  | | | |  | | |
| Scholastic competence | | | |  | | 2302 | | -.043 (-.090, .004) | .024 | .071 | | |  | .049 (.035, .063) | | .007 | **5.4x10^-12^** | |  | -.007 (-.020, .006) | | .007 | .303 | | 3.91% | | | | .65% | | |
| Global self-worth | | | |  | | 2296 | | -.079 (-.126, -.031) | .024 | **.001** | | |  | .048 (.034, .063) | | .007 | **1.8x10^-11^** | |  | -.001 (-.015, .012) | | .007 | .829 | | 4.06% | | | | .82% | | |
| Childhood social skills | | | |  | | 2330 | | -.111 (-.164, -.060) | .026 | **2.4x10^-5^** | | |  | .053 (.039, .067) | | .007 | **1.9x10^-14^** | |  | .016 (.003, .028) | | .006 | **.010** | | 4.63% | | | | .84% | | |
| Adolescent social skills | | | |  | | 2339 | | -.146 (-.198, -.096) | .025 | **5.8x10^-9^** | | |  | .050 (.036, .064) | | .007 | **2.3x10^-12^** | |  | .013 (.001, .027) | | .006 | **.036** | | 5.39% | | | | 2.28% | | |
| Late adolescent social skills | | | |  | | 2092 | | -.120 (-.172, -.068) | .025 | **2.1x10^-6^** | | |  | .043 (.028, .058) | | .007 | **4.8x10^-9^** | |  | .005 (-.009, .019) | | .006 | .435 | | 4.92% | | | | 2.23% | | |
| Academic ability | | | |  | | 2360 | | -.112 (-.158, -.067) | .023 | **1.2x10^-6^** | | |  | .052 (.038, .066) | | .007 | **9.6x10^-14^** | |  | .004 (-.009, .018) | | .007 | .518 | | 4.83% | | | | 1.40% | | |
| **Family-level** | | | |  | |  | |  |  |  | | |  |  | |  |  | |  |  | |  |  | |  | | | |  | | |
| Closeness to parents | | | |  | | 1838 | | -.095 (-.148, -.041) | .027 | **3.9x10^-4^** | | |  | .043 (.028, .060) | | .008 | **3.9x10^-8^** | |  | -.005 (-.020, .010) | | .008 | .517 | | 4.66% | | | | 1.79% | | |
| Closeness to siblings | | | |  | | 1712 | | -.102 (-.157, -.048) | .027 | **1.7x10^-4^** | | |  | .051 (.035, .068) | | .008 | **4.4x10^-10^** | |  | .004 (-.012, .021) | | .008 | .570 | | 4.16% | | | | .77% | | |
| Family support | | | |  | | 1833 | | -.104 (-.154, -.055) | .025 | **3.9x10^-5^** | | |  | .044 (.027, .060) | | .008 | **5.9x10^-8^** | |  | -.004 (-.019, .011) | | .007 | .598 | | 4.53% | | | | 1.65% | | |
| Family involvement | | | |  | | 1824 | | -.094 (-.145, -.043) | .026 | **2.8x10^-4^** | | |  | .049 (.033, .065) | | .008 | **9.5x10^-10^** | |  | -.001 (-.017, .014) | | .008 | .875 | | 4.15% | | | | 1.14% | | |
| Family cohesion | | | |  | | 1838 | | -.102 (-.155, -.049) | .026 | **1.0x10^-4^** | | |  | .042 (.026, .058) | | .008 | **1.9x10^-7^** | |  | -.004 (-.020, .012) | | .008 | .629 | | 4.31% | | | | 1.47% | | |
| **Peer-level** | | | |  | |  | |  |  |  | | |  |  | |  |  | |  |  | |  |  | | | |  | | |  | |
| Childhood friendships | | | |  | | 2303 | | -.067 (-.113, -.021) | .024 | **.005** | | |  | .054 (.039, .069) | | .007 | **2.5x10^-13^** | |  | .007 (-.005, .020) | | .007 | .256 | | 3.73% | | | | .18% | | |
| Adolescent friendships | | | |  | | 2398 | | -.073 (-.118, -.028) | .023 | **.002** | | |  | .049 (.034, .065) | | .007 | **3.3x10^-10^** | |  | .004 (-.008, .016) | | .006 | .506 | | 3.72% | | | | .64% | | |
| Late adolescent friendships | | | |  | | 1811 | | -.171 (-.222, -.119) | .026 | **3.6x10^-11^** | | |  | .049 (.032, .066) | | .008 | **5.2x10^-9^** | |  | .019 (.002, .034) | | .008 | **.015** | | 4.13% | | | | 1.42% | | |
| *Note:*  All models adjusted for sex and socioeconomic status. Results for depressive symptoms were conducted using negative binomial regressions.  R^2^ is the variance accounted for by the main and interactive effects of victimisation and the protective factor, as well as the covariates. ΔR^2^ represents the incremental R^2^. This is the percentage of variance explained by the addition of the protective factor. The ΔR^2^ was calculated by regressing the outcome on victimisation and the covariates, and then including the interaction term with the protective factor and comparing the variance explained. | | | | | | | | | | | | | | | | | | | | | | | | | | | | | | | |

| **Table S8** Impact of victimisation (log-transformed and untransformed), social skills (log-transformed), and their interaction on wellbeing, life satisfaction and depressive symptoms at 23 years | | | | | | | | | | | | | | | | | | | | | | | | | | | | | |
| --- | --- | --- | --- | --- | --- | --- | --- | --- | --- | --- | --- | --- | --- | --- | --- | --- | --- | --- | --- | --- | --- | --- | --- | --- | --- | --- | --- | --- | --- |
|  |  | | **Wellbeing** | | | | | | | | | | | | | | | | | | | | | | | | | | |
|  | |  | | | **Protective Factor** | | | | | |  | | **Victimisation** | | | | | | | |  | **Interaction** | | | | | | |  |
|  | |  | | | **N** | | **β (95% C.I.)** | **SE** | **P value** |  | | | | **β (95% C.I)** | **SE** | **P value** |  | | | **β (95% C.I)** | | | **SE** | | **P value** | | **R^2^** | **ΔR^2^** | |
| **Based on victimisation (log-transformed)** | | | | | | | |  |  | | |  | |  |  |  | |  |  | | | | |  | |  |  |  | |
| Childhood social skills | | | |  | | 2330 | 1.06 (.512, 1.61) | .279 | **1.5x10^-4^** | | |  | | -1.27 (-1.74, -.794) | .241 | **1.6x10^-7^** | |  | -.067 (-.557, .422) | | | | .250 | | .788 | | 3.02% | .96% | |
| Adolescent social skills | | | |  | | 2339 | .850 (.314, 1.39) | .274 | **.002** | | |  | | -1.23 (-1.71, -.758) | .243 | **4.0x10^-7^** | |  | .464 (-.022, .950) | | | | .248 | | .061 | | 3.59% | 1.62% | |
| Late adolescent social skills | | | |  | | 2092 | .853 (.300, 1.41) | .282 | **.003** | | |  | | -1.15 (-1.64, -.647) | .254 | **7.1x10^-6^** | |  | .449 (-.043, .942) | | | | .251 | | .074 | | 3.63% | 1.77% | |
| **Based on victimisation (untransformed)** | | | | | | | |  |  | | |  | |  |  |  | |  |  | | | |  | |  | |  |  | |
| Childhood social skills | | | |  | | 2330 | 1.15 (.679, 1.63) | .242 | **1.9x10^-6^** | | |  | | -.385 (-.522, -.247) | .070 | **5.2x10^-8^** | |  | -.080 (-.208, .049) | | | | .066 | | .226 | | 3.11% | .99% | |
| Adolescent social skills | | | |  | | 2339 | 1.16 (.688, 1.62) | .239 | **1.4x10^-6^** | | |  | | -.373 (-.512, -.233) | .071 | **1.8x10^-7^** | |  | .024 (-.109, .157) | | | | .068 | | .723 | | 3.55% | 1.47% | |
| Late adolescent social skills | | | |  | | 2092 | 1.12 (.644, 1.60) | .244 | **4.5x10^-6^** | | |  | | -.338 (-.481, -.194) | .073 | **4.4x10^-6^** | |  | .044 (-.089, .178) | | | | .068 | | .517 | | 3.57% | 1.66% | |
|  |  | | **Life satisfaction** | | | | | | | | | | | | | | | | | | | | | | | | | | |
| **Based on victimisation (log-transformed)** | | | | | | | |  |  | | |  | |  |  |  | |  |  | | | |  | |  | |  |  | |
| Childhood social skills | | | |  | | 2339 | .837 (.432, 1.24) | .207 | **5.3x10^-5^** | | |  | | -.962 (-1.31, -.613) | .178 | **7.0x10^-8^** | |  | -.156 (-.519, .206) | | | | .184 | | .397 | | 3.32% | 0.99% | |
| Adolescent social skills | | | |  | | 2353 | .760 (.367, 1.15) | .200 | **1.5x10^-4^** | | |  | | -.915 (-1.26, -.567) | .177 | **2.6x10^-7^** | |  | .404 (.048, .760) | | | | .182 | | **.026** | | 4.93% | 2.43% | |
| Late adolescent social skills | | | |  | | 2100 | .724 (.318, 1.13) | .207 | **4.8x10^-4^** | | |  | | -.889 (-1.25, -.524) | .186 | **1.9x10^-6^** | |  | .319 (-.042, .681) | | | | .184 | | .083 | | 4.67% | 2.19% | |
| **Based on victimisation (untransformed)** | | | | | | | |  |  | | |  | |  |  |  | |  |  | | | |  | |  | |  |  | |
| Childhood social skills | | | |  | | 2330 | .906 (.555, 1.26) | .179 | **4.4x10^-7^** | | |  | | -.313 (-.414, -.121) | .051 | **1.6x10^-9^** | |  | -.102 (-.197, -.007) | | | | .049 | | **.036** | | 3.64% | 1.30% | |
| Adolescent social skills | | | |  | | 2339 | .963 (.621, 1.31) | .175 | **3.8x10^-8^** | | |  | | -.284 (-.384, -.182) | .051 | **3.8x10^-8^** | |  | .050 (-.047, .147) | | | | .050 | | .314 | | 4.98% | 2.49% | |
| Late adolescent social skills | | | |  | | 2092 | .917 (.566, 1.27) | .179 | **3.3x10^-7^** | | |  | | -.275 (-.380, -.171) | .053 | **2.5x10^-7^** | |  | .029 (-.069, .126) | | | | .05 | | .563 | | 4.75% | 2.27% | |
|  | | | |  | | **Depressive symptoms** | | | | | | | | | | | | | | | | | | | | | | | |
| **Based on victimisation (log-transformed)** | | | | | | | |  |  | | |  | |  |  |  | |  |  | | | |  | |  | |  |  | |
| Childhood social skills | | | |  | | 2330 | -.096 (-.152, -.040) | .029 | **7.1x10^-4^** | | |  | | .188 (.140, .235) | .024 | **1.4x10^-14^** | |  | .034 (-.016, .084) | | | | .025 | | .171 | | 4.35% | .47% | |
| Adolescent social skills | | | |  | | 2339 | -.121 (-.177, -.067) | .028 | **1.3x10^-5^** | | |  | | .177 (.129, .225) | .025 | **7.2x10^-13^** | |  | .016 (-.033, .066) | | | | .025 | | .510 | | 4.80% | 1.60% | |
| Late adolescent social skills | | | |  | | 2092 | -.101 (-.159, -.044) | .029 | **4.4x10^-4^** | | |  | | .158 (.108, .209) | .026 | **8.9x10^-10^** | |  | -.004 (-.055, .046) | | | | .025 | | .860 | | 4.65% | 1.89% | |
| **Based on victimisation (untransformed)** | | | | | | | |  |  | | |  | |  |  |  | |  |  | | | |  | |  | |  |  | |
| Childhood social skills | | | |  | | 2330 | -.099 (-.148, -.051) | .025 | **5.2x10^-5^** | | |  | | .054 (.040, .068) | .007 | **1.8x10^-14^** | |  | .015 (.002, .029) | | | | .007 | | **.020** | | 4.47% | .68% | |
| Adolescent social skills | | | |  | | 2339 | -.133 (-.181, -.085) | .024 | **3.9x10^-8^** | | |  | | .049 (.035, .064) | .007 | **3.6x10^-12^** | |  | .011 (-.003, .025) | | | | .007 | | .102 | | 4.94% | 1.82% | |
| Late adolescent social skills | | | |  | | 2092 | -.117 (-.167, -.068) | .025 | **2.1x10^-6^** | | |  | | .043 (.028, .058) | .007 | **4.9x10^-9^** | |  | .004 (-.010, .018) | | | | .007 | | .520 | | 4.56% | 1.88% | |
| *Note*:  All models adjusted for sex and socioeconomic status. Results for depressive symptoms were conducted using negative binomial regressions.  R^2^ is the variance accounted for by the main and interactive effects of victimisation and the protective factor, as well as the covariates. ΔR^2^ represents the incremental R^2^. This is the percentage of variance explained by the addition of the protective factor. The ΔR^2^ was calculated by regressing the outcome on victimisation and the covariates, and then including the interaction term with the protective factor and comparing the variance explained. | | | | | | | | | | | | | | | | | | | | | | | | | | | | | |

| **Table S9:** Loadings of principal components on the individual, family, and peer-level protective factors | | | | | |
| --- | --- | --- | --- | --- | --- |
|  | |  | **Loadings** | | |
| **Protective factor** | |  | | **PC1** | **PC2** |
| **Individual-level** |  | | |  |  |
| Scholastic competence |  | | | 0.11 | -0.65 |
| Global self-worth |  | | | 0.14 | -0.60 |
| Childhood social skills |  | | | 0.48 | 0.06 |
| Adolescent social skills |  | | | 0.54 | 0.14 |
| Late adolescent social skills |  | | | 0.48 | 0.12 |
| Academic ability |  | | | 0.15 | 0.13 |
| **Family-level** |  | | |  |  |
| Closeness to parents |  | | | 0.49 | - |
| Closeness to siblings |  | | | 0.41 | - |
| Family support |  | | | 0.47 | - |
| Family involvement |  | | | 0.33 | - |
| Family cohesion |  | | | 0.51 | - |
| **Peer-level** |  | | |  |  |
| Childhood friendships |  | | | -0.49 | - |
| Adolescent friendships |  | | | -0.64 | - |
| Late adolescent friendships |  | | | -0.59 | - |
| **Combined PCA*** |  | | |  |  |
| Individual-level protective factors |  | | | -0.61 | - |
| Family-level protective factors |  | | | 0.59 | - |
| Peer-level protective factors |  | | | -0.52 | - |
| *Note:*  *The combined component was created using a hierarchical PCA of PC1 at the individual-level, family, and peer-level. This component accounted for 41.1% of the variance.  PC1 at the individual-level accounted for 34.7% of the variance, while PC2 accounted for 21.6% of the variance. The family-level component accounted for 52.7% of the variance and the peer-level component accounted for 45.8%. Analyses predicting the individual-level protective factors used factors 1 and 2 to ensure the variance explained was similar to analyses predicting the family and peer-level protective factors. | | | | | |

| **Table S10:** Comparison of wellbeing scores among different protective factor responders in relation to experiences of victimisation | | | | | | | | | | | |  | | |  | |
| --- | --- | --- | --- | --- | --- | --- | --- | --- | --- | --- | --- | --- | --- | --- | --- | --- |
|  |  | | **Never victimised** | |  | **Occasionally victimised** | |  | **Frequently victimised** | |  | | |  | |  |
| **Protective Factor** | | **Total N** | **N (%)** | **Mean (SD)** |  | **N (%)** | **Mean (SD)** |  | **N** | **Mean (SD)** | | ***p1*** | ***p2*** | | | |
| Scholastic competence | | 2302 | 1053 (45.7) | 49.87 (8.73) |  | 857 (37.2) | 48.95 (8.86) |  | 392 (17.0) | 47.38 (9.27) | | <.001 | <.01 | | | |
| Global self-worth | | 2296 | 1050 (45.7) | 49.89 (8.73) |  | 854 (37.2) | 48.97 (8.85) |  | 392 (17.1) | 47.36 (9.26) | | <.001 | <.01 | | | |
| Childhood social skills | | 2330 | 1073 (46.0) | 50.04 (8.75) |  | 864 (37.1) | 48.98 (8.81) |  | 393 (16.9) | 47.45 (9.00) | | <.001 | <.01 | | | |
| Adolescent social skills | | 2339 | 1084 (46.3) | 50.03 (8.77) |  | 867 (37.1) | 48.97 (8.89) |  | 388 (16.6) | 47.38 (9.12) | | <.001 | <.01 | | | |
| Late adolescent social skills | | 2092 | 964 (46.1) | 50.06 (8.76) |  | 778 (37.2) | 48.87 (8.91) |  | 350 (16.7) | 47.70 (8.84) | | <.001 | <.05 | | | |
| Academic ability | | 2360 | 1079 (45.7) | 49.89 (8.75) |  | 877 (37.2) | 48.99 (8.88) |  | 404 (17.1) | 47.36 (9.26) | | <.001 | <.01 | | | |
| Closeness to parents | | 1838 | 849 (46.2) | 50.16 (8.62) |  | 689 (37.5) | 49.38 (8.60) |  | 300 (16.3) | 47.98 (8.34) | | <.001 | <.05 | | | |
| Closeness to siblings | | 1712 | 804 (47.0) | 50.15 (8.58) |  | 633 (37.0) | 49.26 (8.65) |  | 265 (16.1) | 48.08 (8.38) | | <.001 | <.05 | | | |
| Family support | | 1833 | 848 (46.3) | 50.10 (8.63) |  | 688 (37.5) | 49.41 (8.57) |  | 297 (16.2) | 47.98 (8.34) | | <.001 | <.05 | | | |
| Parental involvement | | 1824 | 842 (46.2) | 50.13 (8.63) |  | 686 (37.6) | 49.35 (8.62) |  | 296 (16.2) | 48.11 (8.30) | | <.001 | <.05 | | | |
| Relationship with family | | 1838 | 849 (46.2) | 50.14 (8.61) |  | 689 (37.5) | 49.39 (8.62) |  | 300 (16.3) | 48.00 (8.34) | | <.001 | <.05 | | | |
| Childhood friendships | | 2303 | 1053 (45.7) | 49.96 (8.67) |  | 863 (37.5) | 48.87 (8.91) |  | 387 (16.8) | 47.41 (9.31) | | <.001 | <.01 | | | |
| Adolescent friendships | | 2398 | 1112 (46.4) | 50.01 (8.76) |  | 890 (37.1) | 48.98 (8.83) |  | 396 (16.5) | 47.43 (9.23) | | <.001 | <.01 | | | |
| Late adolescent friendships | | 1811 | 839 (46.3) | 50.12 (8.69) |  | 675 (37.3) | 49.42 (8.54) |  | 297 (16.4) | 48.05 (8.31) | | <.001 | <.05 | | | |
| Participants with complete  data^a^ | | 949 | 443 (46.7) | 50.28 (8.52) |  | 368 (38.7) | 49.75 (8.52) |  | 138 (14.5) | 49.33 (8.13) | | .23 | .37 | | | |
| *Note:*  ^a^ Participants had complete data on all protective factor measures, as well as the victimisation scale, the wellbeing and depression assessments, and SES.  N represents the number of participants who had complete data on the protective factor measure, the victimisation scale, the wellbeing and depression assessments, and SES.  *p1* represents the t-test results comparing the mean wellbeing scores of those never victimised to those frequently victimised.  *p2* represents the t-test results comparing the mean wellbeing scores of those occasionally victimised to those frequently victimised. | | | | | | | | | | | | | | | |  |

| **Table S11:** Impact of victimisation (log-transformed), protective factors, and their interaction on wellbeing at 23 years (full results) | | | | | | | | | | | | | | | | | | | | | | | | | | | | | | |
| --- | --- | --- | --- | --- | --- | --- | --- | --- | --- | --- | --- | --- | --- | --- | --- | --- | --- | --- | --- | --- | --- | --- | --- | --- | --- | --- | --- | --- | --- | --- |
|  |  | |  | | | | **Wellbeing** | | | | | | | | | | | | | | | | | | | |  | |  |  |
|  | |  | | | **Protective Factor** | | | | | | |  | | | | **Victimisation** | | |  | | | **Interaction** | | | | | | | | |
|  | |  | | | **N** | | | **β (95% C.I.)** | **SE** | **P value** |  | | | | **β (95% C.I)** | | **SE** | **P value** | |  | | **β (95% C.I)** | **SE** | **P value** | | **R^2^** | | **ΔR^2^** | | |
| **Individual-level** | | | |  | |  | |  |  |  | | |  |  | | |  |  | |  | |  |  | |  |  | |  | | |
| Scholastic competence | | | |  | | 2302 | | .099 (-.422, .619) | .265 | .721 | | |  | -1.31 (-1.78, -.831) | | | .243 | **8.2x10^-8^** | |  | .631 (.152, 1.10) | | .244 | **.010** | | 2.50% | | .58% | | |
| Global self-worth | | | |  | | 2296 | | .690 (.162, 1.22) | .269 | **.010** | | |  | -1.26 (-1.74, -.782) | | | .243 | **2.5x10^-7^** | |  | .254 (-.218, .727) | | .241 | .291 | | 2.87% | | .88% | | |
| Childhood social skills | | | |  | | 2330 | | 1.16 (.562, 1.75) | .303 | **1.4x10^-5^** | | |  | -1.26 (-1.74, .792) | | | .241 | **1.7x10^-7^** | |  | -.049 (-.549, .452) | | .255 | .849 | | 3.08% | | 1.02% | | |
| Adolescent social skills | | | |  | | 2339 | | .952 (.384, 1.52) | .290 | **.001** | | |  | -1.26 (-1.73, -.781) | | | .243 | **2.4x10^-7^** | |  | .393 (-.094, .880) | | .248 | .114 | | 3.62% | | 1.65% | | |
| Late adolescent social skills | | | |  | | 2092 | | .995 (.417, 1.57) | .295 | **7.6x10^-4^** | | |  | -1.14 (-1.63, -.637) | | | .254 | **8.3x10^-6^** | |  | .379 (-.109, .867) | | .249 | .128 | | 3.86% | | 2.00% | | |
| Academic ability | | | |  | | 2360 | | 1.30 (.081, 1.80) | .254 | **3.2x10^-7^** | | |  | -1.26 (-1.72, -.079) | | | .238 | **1.4x10^-7^** | |  | .161 (-.296, .618) | | .233 | .490 | | 4.37% | | 2.35% | | |
| **Family-level** | | | |  | |  | |  |  |  | | |  |  | | |  |  | |  |  | |  |  | |  | |  | | |
| Closeness to parents | | | |  | | 1838 | | 1.34 (.761, 1.93) | .297 | **6.5x10^-6^** | | |  | -1.05 (-1.56, -.532) | | | .263 | **7.1x10^-5^** | |  | .007 (-.520, .535) | | .269 | .978 | | 3.67% | | 2.09% | | |
| Closeness to siblings | | | |  | | 1712 | | 1.61 (1.02, 2.19) | .299 | **2.1x10^-8^** | | |  | -1.09 (-1.62, -.550) | | | .273 | **7.4x10^-5^** | |  | -.405 (-.955, .146) | | .281 | .150 | | 3.72% | | 2.11% | | |
| Family support | | | |  | | 1833 | | 1.52 (.970, 2.06) | .278 | **5.8x10^-8^** | | |  | -1.02 (-1.54, -.509) | | | .263 | **1.0x10^-4^** | |  | .004 (-.523, .513) | | .264 | .985 | | 4.33% | | 2.78% | | |
| Family involvement | | | |  | | 1824 | | .740 (.188, 1.29) | .281 | **.009** | | |  | -1.08 (-1.60, -.556) | | | .266 | **5.4x10^-5^** | |  | .402 (-.118, .922) | | .265 | .130 | | 2.78% | | 1.35% | | |
| Family cohesion | | | |  | | 1838 | | 1.35 (.776, 1.93) | .293 | **1.5x10^-6^** | | |  | -.900 (-1.42, -.382) | | | .264 | **6.7x10^-4^** | |  | .108 (-.406, .622) | | .262 | .681 | | 4.06% | | 2.48% | | |
| **Peer-level** | | | |  | |  | |  |  |  | | |  |  | | |  |  | |  |  | |  |  | |  | |  | | |
| Childhood friendships | | | |  | | 2303 | | .616 (.082, 1.15) | .272 | **.024** | | |  | -1.31 (-1.79, -.827) | | | .246 | **1.2x10^-7^** | |  | -.052 (-.528, .424) | | .243 | .831 | | 2.53% | | .30% | | |
| Adolescent friendships | | | |  | | 2398 | | .913 (.392, 1.43) | .266 | **6.0x10^-4^** | | |  | -1.38 (-1.63, -.648) | | | .250 | **5.6x10^-6^** | |  | .040 (-.395, .475) | | .222 | .856 | | 2.97% | | .98% | | |
| Late adolescent friendships | | | |  | | 1811 | | 2.46 (1.92, 3.01) | .277 | **2.0x10^-16^** | | |  | -.976 (-1.51, -.445) | | | .271 | **3.2x10^-4^** | |  | -.745 (-1.25, -.237) | | .259 | **.004** | | 6.81% | | 5.24% | | |
| *Note:*  All models adjusted for sex and socioeconomic status.  R^2^ is the variance accounted for by the main and interactive effects of victimisation and the protective factor, as well as the covariates. ΔR^2^ represents the incremental R^2^. This is the percentage of variance explained by the addition of the protective factor. The ΔR^2^ was calculated by regressing the outcome on victimisation and the covariates, and then including the interaction term with the protective factor and comparing the variance explained. | | | | | | | | | | | | | | | | | | | | | | | | | | | | | | |

| **Table S12:** Impact of victimisation (log-transformed), protective factors, and their interaction on wellbeing at 23 years (imputed dataset) | | | | | | | | | | | | | | | | | | | | | | | | | | | | | | | | | | | | | |  |  | | | | |  |
| --- | --- | --- | --- | --- | --- | --- | --- | --- | --- | --- | --- | --- | --- | --- | --- | --- | --- | --- | --- | --- | --- | --- | --- | --- | --- | --- | --- | --- | --- | --- | --- | --- | --- | --- | --- | --- | --- | --- | --- | --- | --- | --- | --- | --- |
|  |  | **Wellbeing** | | | | | | | | | | | | | | | | | | | | | | | | | | | | | | | | | | | |  |  | | | | |  |
|  | | | | **Protective Factor** | | | | | | | | |  | | | **Victimisation** | | | | | | | | | | | | | **Interaction** | | | | | | | | | | | | | | |  |
|  | | | | **β (95% C.I.)** | **SE** | | | | **P value** | |  | | | | | | | **β (95% C.I)** | | | **SE** | | **P value** | |  | | | | | | **β (95% C.I)** | | **SE** | **P value** | | | **R^2^** | | | | | **ΔR^2^** | |  |
| **Individual-level** | | |  | | |  | |  | | | |  | | |  | | | |  | | |  | | | |  | |  | | | |  | | |  |  | | | |  | | |  |  |
| Scholastic competence | | | .482 (.101, .866) | | | | .194 | | | **.013** | | | |  | | | -1.15 (-1.51, -.781) | | | .187 | | | | **9.4x10^-10^** | | |  | | | .448 (.089, .807) | | | .183 | **.015*** | | | 2.59% | | | | .94% | | | |
| Global self-worth | | | .841 (.453, 1.23) | | | | .198 | | | **2.2x10^-5^** | | | |  | | | -1.08 (-1.45, -.717) | | | .187 | | | | **7.6x10^-10^** | | |  | | | .188 (-.166, .543) | | | .181 | .297 | | | 2.84% | | | | 1.19% | | | |
| Childhood social skills | | | 2.65 (1.95, 3.35) | | | | .359 | | | **1.8x10^13^** | | | |  | | | -1.12 (-1.49, -.756) | | | .187 | | | | **1.9x10^-9^** | | |  | | | -.167 (-.540, .208) | | | .190 | .385 | | | 2.95% | | | | 1.31% | | | |
| Adolescent social skills | | | .886 (.499, 1.27) | | | | .197 | | | **7.3x10^-6^** | | | |  | | | -1.07 (-1.44, -.703) | | | .187 | | | | **1.2x10^-8^** | | |  | | | .162 (-.192, .515) | | | .180 | .371 | | | 2.79% | | | | 1.16% | | | |
| Late adolescent social skills | | | .717 (.316, 1.12) | | | | .204 | | | **4.5x10^-4^** | | | |  | | | -.985 (-1.35, -.618) | | | .187 | | | | **1.5x10^-5^** | | |  | | | .666 (.305, 1.03) | | | .184 | **3.0x10^-4^** | | | 3.57% | | | | 1.93% | | | |
| Academic ability | | | 1.32 (.940, 1.70) | | | | .195 | | | **1.4x10^-11^** | | | |  | | | -1.10 (-1.47, -.740) | | | .186 | | | | **2.9x10^-9^** | | |  | | | .087 (-.270, .445) | | | .182 | .632 | | | 3.93% | | | | 2.30% | | | |
| **Family-level** | | |  | | | |  | | |  | | | |  | | |  | | |  | | | |  | | |  | | |  | | |  |  | | |  | | | |  | | | |
| Closeness to parents | | | 1.16 (.781, 1.54) | | | | .194 | | | **2.3x10^-9^** | | | |  | | | -1.12 (-1.48, -.755) | | | .185 | | | | **1.7x10^-9^** | | |  | | | .376 (.022, .729) | | | .181 | **.037** | | | 4.27% | | | | 2.63% | | | |
| Closeness to siblings | | | 1.25 (.870, 1.62) | | | | .192 | | | **9.1x10^-11^** | | | |  | | | -1.16 (-1.52, -.798) | | | .185 | | | | **3.9x10^-10^** | | |  | | | .127 (-.227, .482) | | | .181 | .482 | | | 3.82% | | | | 2.18% | | | |
| Family support | | | 1.59 (1.22, 1.96) | | | | .191 | | | **2.0x10^-16^** | | | |  | | | -1.12 (-1.48, -.755) | | | .184 | | | | **1.54x10^-9^** | | |  | | | .129 (-.223, .481) | | | .180 | .473 | | | 5.09% | | | | 3.45% | | | |
| Family involvement | | | .702 (.319, 1.09) | | | | .196 | | | **3.4x10^-4^** | | | |  | | | -1.18 (-1.54, -.811) | | | .186 | | | | **2.9x10^-10^** | | |  | | | .364 (.007, .721) | | | .182 | **.045** | | | 2.88% | | | | 1.24% | | | |
| Family cohesion | | | 1.22 (.838, 1.60) | | | | .194 | | | **3.1x10^-10^** | | | |  | | | -.937 (-1.30, -.573) | | | .186 | | | | **4.9x10^-7^** | | |  | | | .471 (.117, .825) | | | .180 | **.009** | | | 4.74% | | | | 3.10% | | | |
| **Peer-level** | | |  | | | |  | | |  | | | |  | | |  | | |  | | | |  | | |  | | |  | | |  |  | | |  | | | |  | | | |
| Childhood friendships | | | .492 (.101, .883) | | | | .200 | | | **.014** | | | |  | | | -1.14 (-1.51, -.767) | | | .189 | | | | **1.9x10^-9^** | | |  | | | -.030 (-.389, .330) | | | .184 | .872 | | | 1.91% | | | | .26% | | | |
| Adolescent friendships | | | .886 (.486, 1.29) | | | | .204 | | | **1.5x10^-5^** | | | |  | | | -1.02 (-1.40, -.646) | | | .193 | | | | **1.1x10^-7^** | | |  | | | -.172 (-.512, .169) | | | .174 | .323 | | | 2.30% | | | | .65% | | | |
| Late adolescent friendships | | | 2.02 (1.65, 2.40) | | | | .191 | | | **2.0x10^-16^** | | | |  | | | -.978 (-1.34, -.615) | | | .185 | | | | **1.3x10^-7^** | | |  | | | -.180 (-.523, .163) | | | .175 | .302 | | | 5.93% | | | | 4.28% | | | |
| *Note*:  Imputed dataset n=4044. All models adjusted for sex and socioeconomic status.  R^2^ is the variance accounted for by the main and interactive effects of victimisation and the protective factor, as well as the covariates. ΔR^2^ represents the incremental R^2^. This is the percentage of variance explained by the addition of the protective factor. The ΔR^2^ was calculated by regressing the outcome on victimisation and the covariates, and then including the interaction term with the protective factor and comparing the variance explained. | | | | | | | | | | | | | | | | | | | | | | | | | | | | | | | | | | | | | | | | | | | | |

| **Table S13:** Impact of victimisation (log-transformed), protective factors, and their interaction on life satisfaction and depressive symptoms at 23 years | | | | | | | | | | | | | | | | | | | | | | | | | | | | | | | | | | | |
| --- | --- | --- | --- | --- | --- | --- | --- | --- | --- | --- | --- | --- | --- | --- | --- | --- | --- | --- | --- | --- | --- | --- | --- | --- | --- | --- | --- | --- | --- | --- | --- | --- | --- | --- | --- |
|  |  | |  | | | | **Impact on life satisfaction using log-transformed victimisation scores** | | | | | | | | | | | | | | | | | | | | | | |  | |  | | |  |
|  | |  | | |  | | | **Protective Factor** | | | |  | | | | **Victimisation** | | |  | | | | | | **Interaction** | | | | | | | | | |  |
|  | |  | | | **N** | | | | **β (95% C.I.)** | **SE** | **P value** | |  | | **β (95% C.I)** | | **SE** | **P value** | |  | | | | **β (95% C.I)** | | **SE** | **P value** | | **R^2^** | | | | **ΔR^2^** | | |
| **Individual-level** | | | |  | |  | | |  |  |  | | |  |  | |  |  | |  | |  | | | |  | |  |  | | | |  | | |
| Scholastic competence | | | |  | | 2310 | | | .348 (-.037, .733) | .196 | .076 | | |  | -1.04 (-1.39, -.692) | | .179 | **6.5x10^-9^**† | |  | | .169 (-.181, .519) | | | | .179 | .343 | | 2.81% | | | | .43% | | |
| Global self-worth | | | |  | | 2304 | | | .551 (.160, .943) | .200 | **.006**† | | |  | -1.02 (-1.37, -.670) | | .179 | **1.3x10^-8^**† | |  | | .168 (-.181, .517) | | | | .178 | .345 | | 3.39% | | | | .94% | | |
| Childhood social skills | | | |  | | 2339 | | | .954 (.513, 1.39) | .225 | **2.3x10^-5^**† | | |  | -.953 (-1.30, -.604) | | .178 | **9.1x10^-8^**† | |  | | -.137 (-.508, .235) | | | | .189 | .471 | | 3.28% | | | | 1.07% | | |
| Adolescent social skills | | | |  | | 2353 | | | .815 (.400, 1.23) | .212 | **1.2x10^-4^**† | | |  | -.940 (-1.29, -.593) | | .212 | **1.2x10^-7^**† | |  | | .353 (-.004, .711) | | | | .182 | .053 | | 4.68% | | | | 2.31% | | |
| Late adolescent social skills | | | |  | | 2100 | | | .760 (.333, 1.19) | .218 | **4.8x10^-4^**† | | |  | -.887 (-1.25, -.522) | | .186 | **1.9x10^-6^**† | |  | | .322 (-.037, .681) | | | | .183 | .079 | | 4.63% | | | | 2.29% | | |
| Academic ability | | | |  | | 2370 | | | .991 (.621, 1.36) | .189 | **1.6x10^-7^**† | | |  | -1.00 (-1.35, -.661) | | .175 | **1.1x10^-8^**† | |  | | -.057 (-.395, .281) | | | | .173 | .741 | | 4.15% | | | | 1.61% | | |
| **Family-level** | | | |  | |  | | |  |  |  | | |  |  | |  |  | |  | |  | | | |  |  | |  | | | |  | | |
| Closeness to parents | | | |  | | 1850 | | | 1.14 (.702, 1.58) | .223 | **3.4x10^-7^**† | | |  | -.850 (-1.23, -.465) | | .196 | **1.6x10^-5^**† | |  | | -.147 (-.541, .246) | | | | .201 | .463 | | 3.91% | | | | 1.80% | | |
| Closeness to siblings | | | |  | | 1722 | | | .854 (.410, 1.30) | .226 | **1.6x10^-4^**† | | |  | -.952 (-1.35, -.551) | | .204 | **3.4x10^-6^**† | |  | | -.142 (-.555, .271) | | | | .211 | .500 | | 2.70% | | | | .70% | | |
| Family support | | | |  | | 1845 | | | .888 (.478, 1.30) | .209 | **2.3x10^-5^**† | | |  | -.837 (-1.22, -.452) | | .196 | **2.1x10^-5^**† | |  | | .218 (-.169, .605) | | | | .197 | .269 | | 4.10% | | | | 1.97% | | |
| Family involvement | | | |  | | 1836 | | | .683 (.272, 1.09) | .210 | **.001**† | | |  | -.890 (-1.28, -.502) | | .198 | **7.2x10^-6^**† | |  | | .244 (-.141, .628) | | | | .196 | .214 | | 3.28% | | | | 1.34% | | |
| Family cohesion | | | |  | | 1850 | | | 1.11 (.680, 1.55) | .198 | **5.1x10^-7^**† | | |  | -.747 (-1.13, -.360) | | .198 | **1.6x10^-4^**† | |  | | -.038 (-.424, .348) | | | | .197 | .847 | | 4.22% | | | | 2.07% | | |
| **Peer-level** | | | |  | |  | | |  |  |  | | |  |  | |  |  | |  | |  | | | |  |  | |  | | | |  | | |
| Childhood friendships | | | |  | | 2313 | | | .425 (.028, .822) | .202 | **.036** | | |  | -1.08 (-1.44, -.721) | | .183 | **3.9x10^-9^**† | |  | | -.127 (-.480, .225) | | | | .180 | .478 | | 2.61% | | | | .12% | | |
| Adolescent friendships | | | |  | | 2408 | | | .539 (.153, .925) | .197 | **.006**† | | |  | -.882 (-1.24, -.520) | | .184 | **1.8x10^-6^**† | |  | | .081 (-.239, .401) | | | | .163 | .619 | | 2.89% | | | | .44% | | |
| Late adolescent friendships | | | |  | | 1823 | | | 1.45 (1.04, 1.86) | .208 | **4.7x10^-12^**† | | |  | -.800 (-1.19, -.412) | | .198 | **5.5x10^-5^**† | |  | | -.324 (-.698, .051) | | | | .191 | .090 | | 5.11% | | | | 2.96% | | |
|  |  | |  | | | | **Impact on depressive symptoms using log-transformed victimisation scores** | | | | | | | | | | | | | | | | | | | | | |  | | | |  | | |
| **Individual-level** | | | |  | |  | | |  |  |  | | |  |  | |  |  | |  | |  | | | |  |  | |  | | | |  | | |
| Scholastic competence | | | |  | | 2302 | | | -.036 (-.089, .018) | .027 | .189 | | |  | .178 (.130, .226) | | .024 | **3.8x10^-13^**† | | |  | | -.030 (-.076, .017) | | | .025 | .229 | | 4.01% | | | | .63% | | |
| Global self-worth | | | |  | | 2296 | | | -.071 (-1.25, -.017) | .028 | **.010**† | | |  | .172 (.125, .220) | | .025 | **2.3x10^-12^**† | | |  | | -.016 (-.064, .032) | | | .024 | .513 | | 4.08% | | | | .92% | | |
| Childhood social skills | | | |  | | 2330 | | | -.109 (-.171, .048) | .031 | **3.9x10^-4^**† | | |  | .187 (.140, .234) | | .024 | **1.7x10^-14^**† | | |  | | .040 (-.013, .091) | | | .026 | .121 | | 4.48% | | | | .59% | | |
| Adolescent social skills | | | |  | | 2339 | | | -.134 (-.194, -.076) | .029 | **4.5x10^-5^**† | | |  | .178 (.130, .226) | | .025 | **4.8x10^-13^**† | | |  | | .023 (-.029, .075) | | | .025 | .347 | | 5.17% | | | | 1.96% | | |
| Late adolescent social skills | | | |  | | 2092 | | | -.101 (-.162, -.040) | .030 | **7.4x10^-4^**† | | |  | .158 (.108, .208) | | .026 | **9.1x10^-10^**† | | |  | | -.005 (-.056, .046) | | | .025 | .852 | | 4.96% | | | | 2.21% | | |
| Academic ability | | | |  | | 2360 | | | -.114 (-.165, -.064) | .026 | **1.4x10^-5^**† | | |  | .187 (.140, .235) | | .024 | **1.1x10^-14^**† | | |  | | .012 (-.034, .058) | | | .024 | .623 | | 5.01% | | | | 1.54% | | |
| **Family-level** | | | |  | |  | | |  |  |  | | |  |  | |  |  | | |  | |  | | |  |  | |  | | | |  | | |
| Closeness to parents | | | |  | | 1838 | | | -.092 (-.154, -.031) | .031 | **.003**† | | |  | .158 (.105, .211) | | .027 | **5.8x10^-9^**† | | |  | | -.018 (-.071, .036) | | | .027 | .524 | | 4.45% | | | | 1.59% | | |
| Closeness to siblings | | | |  | | 1712 | | | -.100 (-.162, -.039) | .031 | **3.9x10^-4^**† | | |  | .180 (.125, .235) | | .028 | **1.8x10^-10^**† | | |  | | .011 (-.045, .067) | | | .029 | .701 | | 4.09% | | | | .73% | | |
| Family support | | | |  | | 1833 | | | -.108 (-.165, -.052) | .029 | **5.1x10^-4^**† | | |  | .161 (.108, .215) | | .027 | **3.3x10^-9^**† | | |  | | -.009 (-.062, .043) | | | .027 | .735 | | 4.57% | | | | 1.71% | | |
| Family involvement | | | |  | | 1824 | | | -.088 (-.145, -.032) | .029 | **2.5x10^-4^**† | | |  | .172 (.119, .225) | | .027 | **3.2x10^-10^**† | | |  | | -.011 (-.064, .041) | | | .027 | .680 | | 4.13% | | | | 1.21% | | |
| Family cohesion | | | |  | | 1838 | | | -.101 (-.162, -.041) | .030 | **9.0x10^-4^**† | | |  | .150 (.097, .204) | | .027 | **3.9x10^-8^**† | | |  | | -.011 (-.064, .042) | | | .027 | .693 | | 4.24% | | | | 1.43% | | |
| **Peer-level** | | | |  | |  | | |  |  |  | | |  |  | |  |  | | |  | |  | | |  |  | | | |  | | |  | |
| Childhood friendships | | | |  | | 2303 | | | -.063 (-.118, -.009) | .028 | **.024**† | | |  | .186 (.137, .234) | | .025 | **1.3x10^-13^**† | | |  | | .015 (-.033, .062) | | | .025 | .552 | | 3.77% | | | | .28% | | |
| Adolescent friendships | | | |  | | 2398 | | | -.060 (-.112, -.008) | .027 | **.029**† | | |  | .165 (.116, .215) | | .025 | **7.1x10^-11^**† | | |  | | -.003 (-.046, .039) | | | .022 | .890 | | 3.82% | | | | .63% | | |
| Late adolescent friendships | | | |  | | 1811 | | | -.176 (-.233, -.118) | .029 | **1.9x10^-9^**† | | |  | .163 (.108, .218) | | .028 | **1.1x10^-8^**† | | |  | | .054 (-.005, .109) | | | .027 | **.044** | | 4.13% | | | | 1.55% | | |
| *Note:*  All models adjusted for sex and socioeconomic status. Results for depressive symptoms were conducted using negative binomial regressions. †FDR  R^2^ is the variance accounted for by the main and interactive effects of victimisation and the protective factor, as well as the covariates. ΔR^2^ represents the incremental R^2^. This is the percentage of variance explained by the addition of the protective factor. The ΔR^2^ was calculated by regressing the outcome on victimisation and the covariates, and then including the interaction term with the protective factor and comparing the variance explained. | | | | | | | | | | | | | | | | | | | | | | | | | | | | | | | | | | | |

| **Table S14:** Impact of victimisation (log-transformed), principal components, and their interaction on wellbeing at 23 years | | | | | | | | | |  |  |
| --- | --- | --- | --- | --- | --- | --- | --- | --- | --- | --- | --- |
|  | |  | **Wellbeing** | | | | | |  | | |
|  | |  | **Protective factor (PC)** | **Victimisation** | **Interaction** | |  | |  | | |
|  | **N** | | **β (95%C.I.)** | **β (95%C.I.)** | **β (95%C.I.)** | **ΔR^2^** | | | |  |  |
| **Individual-level** |  | |  |  |  |  | |  |  |  |  |
| PC1 | 1571 | | **.95 (.33,.1.6)**** | **-1.0 (-1.6,-.43)***** | **.94 (.37,1.5)**** | 4.1% | | | | |  |
| PC2 | 1571 | | .52 (-.09,1.1) | **-1.4 (-1.9,-.82)***** | -.00 (-.54,.54) | 0.3% | | | | |  |
| **Family-level** |  | |  |  |  |  | | | | |  |
| PC1 | 1663 | | **1.7 (1.1,2.3)***** | **-.97 (-1.5,-.43)***** | .09 (-.43,.61) | 4.2% | | | | |  |
| **Peer-level** |  | |  |  |  |  | | | | |  |
| PC1 | 1476 | | **-1.9 (-2.5,-1.3)***** | **-.70 (-1.3,-.10)*** | .32 (-.25,.89) | 3.6% | | | | |  |
| **Combined** |  | |  |  |  |  | | | | |  |
| PC1 | 939 | | **-2.3 (-3.1, -1.5)***** | -.03 (-.78,.72) | -.09 (-.80,.63) | 7.2% | | | | |  |
| *Note:*  ΔR^2^ represents the incremental R^2.^ The combined component was created using a hierarchical PCA of PC1 at the individual-level, family, and peer-level.  ***p<0.001, **p<0.01, *p<0.05*.* | | | | | | | | | |  |  |

| **Table S15:** Impact of victimisation (log-transformed), principal components, and their interaction on life satisfaction and depressive symptoms | | | | | | | | | | | | | | | | | | | | | | |
| --- | --- | --- | --- | --- | --- | --- | --- | --- | --- | --- | --- | --- | --- | --- | --- | --- | --- | --- | --- | --- | --- | --- |
|  |  | | **Life satisfaction** | | | | | | |  | | | **Depressive symptoms** | | | | | | | | | |
|  |  | | **Protective factor (PC)** | | **Victimisation** | **Interaction** | |  | |  | | | **Protective factor (PC)** | | | | **Victimisation** | | **Interaction** | | | |
|  | | **N** | | **β (95%C.I.)** | **β (95%C.I.)** | **β (95%C.I.)** | **ΔR^2^** | | | |  |  | | | **β (95%C.I.)** | | **β (95%C.I.)** | | **β (95%C.I.)** | **ΔR^2^** | |  |
| **Individual-level** | |  | |  |  |  |  | |  | | | | | | |  | |  |  |  |  |  |
| PC1 | | 1571 | | **.82 (.38,1.3)***** | **-.67 (-1.1,-.25)***** | **.70 (.29,1.1)**** | 4.9% | | | | | | | **-.11 (-.17,-.05)***** | | | **.16 (.10,.22)***** | | -.03 (-.09,.03) | | 3.9% | |
| PC2 | | 1571 | | .41 (-.04,.86) | **-1.0 (-1.4,-.58)***** | -.13 (-.53,.26) | .25% | | | | | | | **-.06 (-.12,.00)** | | | **.19 (.14,.25)***** | | .01 (-.04,.06) | | 0.1% | |
| **Family-level** | |  | |  |  |  |  | | | | | | |  | | |  | |  | |  | |
| PC1 | | 1663 | | **1.2 (.80,1.7)***** | **-.78 (-1.2,-.38)***** | .07 (-.32,.46) | 3.9% | | | | | | | **-.12(-.18,-.06)***** | | | **.16 (.11,.22)***** | | -.02 (-.08,.03) | | 2.2% | |
| **Peer-level** | |  | |  |  |  |  | | | | | | |  | | |  | |  | |  | |
| PC1 | | 1476 | | **-1.1 (-1.6,-.64)***** | **-.55 (-1.0,-.10)*** | .07 (-.36,.49) | 2.5% | | | | | | | **.13 (.06,.19)***** | | | **.12 (.06,.19)***** | | -.01 (-.07,.05) | | 1.2% | |
| **Combined** | |  | |  |  |  |  | | | | | | |  | | |  | |  | |  | |
| PC1 | | 939 | | **-1.8 (-2.3,-1.2)***** | -.03 (-.58,.51) | -.19 (-.33,.71) | 6.5% | | | | | | | **.15 (.08,.23)***** | | | **.09 (.01,.18)*** | | -.00 (-.07,.07) | | 3.1% | |
| *Note:*  ΔR^2^ represents the incremental R^2.^ The combined component was created using a hierarchical PCA of PC1 at the individual-level, family, and peer-level.  ***p<0.001, **p<0.01, *p<0.05*.* | | | | | | | | | | | | | | | | | | | | | | |

**References**

Faul, F., Erdfelder, E., Buchner, A., & Lang, A-G. (2009). Statistical power analyses using G*Power 3.1: Tests for correlation and regression analyses. Behavior Research Methods, 41(4), 1149-1160.

Flaspohler, P., Elfstrom, J., Vanderzee, K., Sink, H., & Birchmeier, Z. (2009). Stand by me: The effects of peer and teacher support in mitigating impact of bullying on quality of life. *Psychology in the Schools, 46,* 636-649.

Perugini, M., Gallucci, M., & Costantini, G. (2018). A Practical Primer To Power Analysis for Simple Experimental Designs*. International Review of Social Psychology, 31*(1), 20. DOI: http://doi.org/10.5334/irsp.181
